# Leucine/glutamine and v-ATPase/lysosomal acidification via mTORC1 activation are required for position-dependent regeneration

**DOI:** 10.1038/s41598-018-26664-2

**Published:** 2018-05-29

**Authors:** Kazuya Takayama, Akihiko Muto, Yutaka Kikuchi

**Affiliations:** 1 Department of Biological Science, Graduate School of Science, Hiroshima University, Kagamiyama 1-3-1, Higashi-Hiroshima, Hiroshima, 739-8526 Japan; 2 Present Address: Hematology Business Development, HU Business Development, Sysmex Corporation, 4-4-4 Takatsukadai, Nishi-ku, Kobe 651-2271 Japan

## Abstract

In animal regeneration, control of position-dependent cell proliferation is crucial for the complete restoration of patterned appendages in terms of both, shape and size. However, detailed mechanisms of this process are largely unknown. In this study, we identified leucine/glutamine and v-ATPase/lysosomal acidification, via mechanistic target of rapamycin complex 1 (mTORC1) activation, as effectors of amputation plane-dependent zebrafish caudal fin regeneration. mTORC1 activation, which functions in cell proliferation, was regulated by lysosomal acidification possibly via v-ATPase activity at 3 h post amputation (hpa). Inhibition of lysosomal acidification resulted in reduced growth factor-related gene expression and suppression of blastema formation at 24 and 48 hpa, respectively. Along the proximal-distal axis, position-dependent lysosomal acidification and mTORC1 activation were observed from 3 hpa. We also report that Slc7a5 (L-type amino acid transporter), whose gene expression is position-dependent, is necessary for mTORC1 activation upstream of lysosomal acidification during fin regeneration. Furthermore, treatment with leucine and glutamine, for both proximal and distal fin stumps, led to an up-regulation in cell proliferation via mTORC1 activation, indicating that leucine/glutamine signaling possesses the ability to change the position-dependent regeneration. Our findings reveal that leucine/glutamine and v-ATPase/lysosomal acidification via mTORC1 activation are required for position-dependent zebrafish fin regeneration.

## Introduction

Complete regeneration (in terms of both shape and size) of the patterned appendages is observed in some vertebrates such as fish and salamanders^1,2^. This restorative ability is achieved via cellular properties that control cell proliferation and patterning depending on regional-specific information, also known as positional memory^3–6^. To date, three factors (transmembrane receptor Prod1^7,8^, retinoic acid (RA)^5^, and Hand2^9^) have been proposed as effectors of the positional memory in salamander limbs, amphibian limbs, and zebrafish pectoral fins, respectively. The expression of *Prod1* and *hand2* exhibits a gradient along the proximal-distal (P-D) axis in salamander limbs^8^ and zebrafish pectoral fins^9^, respectively, while no gradient of the RA signaling components has been reported in salamander limbs or zebrafish fins prior to injury. Although a recent report identified many genes, proteins, and metabolites via omics analyses in addition to Prod1, RA, and Hand2 along the P-D axis of the zebrafish caudal fin^10^, the detailed molecular regulatory mechanisms of position-dependent regeneration are still largely unknown.

Signaling molecules, such as transforming growth factor-β (TGF–β), Wnt, fibroblast growth factor (FGF), Notch, Hedgehog, insulin-like growth factor (IGF), RA, and mechanistic target of rapamycin (mTOR) have been determined to be necessary for appendage regeneration^11,12^. Among them, the mTOR is well known to sense environmental cues (growth factors, nutrients, and the cellular energy change) and control the cell growth and metabolism^13^. mTOR belongs to a serine/threonine protein kinase family and exists as two distinct complexes-mTOR complex1 (mTORC1) and 2 (mTORC2)^13^. In the mTORC1 signaling pathway, growth factors, intracellular and environmental stresses (e.g. energy, oxygen, and DNA damage), and amino acids are known to be upstream regulators^14,15^. mTORC1 regulates cell growth via protein, lipid, and nucleotide synthesis, and it is also known to inhibit autophagy^14,15^. The amino acids, especially leucine, glutamine, and arginine, activate mTORC1 signaling via a lysosomal amino acid transporter (SLC38A9) and a RAS-related GTP-binding protein (Rag)/Regulator/vacuolar-type proton transporter H^+^-ATPase (v-ATPase) complex^14,15^. A previous report proved that the expression of v-ATPase demonstrated position-dependency and that the v-ATPase activity was necessary for the expression of *aldehyde dehydrogenase 1 family, member A2* (*aldh1a2*) (a type of RA synthetase) during zebrafish caudal fin regeneration^16^. However, the function of the amino acids and SLC38A9/Rag/Regulator/v-ATPase complex in the position-dependent appendage regeneration process has not yet been reported.

Zebrafish caudal fins are complex appendages that are composed of epidermal cells, segmented bony rays, blood vessels, fibroblast-like mesenchymal cells, and nerve axons. Using zebrafish caudal fin regeneration systems, we have previously showed that mTORC1 signaling regulated cell proliferation, cell survival, and differentiation in zebrafish fin regeneration downstream of the IGF and Wnt pathways^17^. However, the regulation of mTORC1 activity via upstream regulators other than growth factors during fin regeneration has not yet been analyzed. In this study, we explored the upstream regulators of mTORC1 and identified the axis of leucine/glutamine signaling-v-ATPase/lysosomal acidification via mTORC1 activation as an effector of position-dependent zebrafish caudal fin regeneration.

## Results

### mTORC1 activation is not controlled by IGF and Wnt signaling at 3 h post amputation

**Figure 1:**
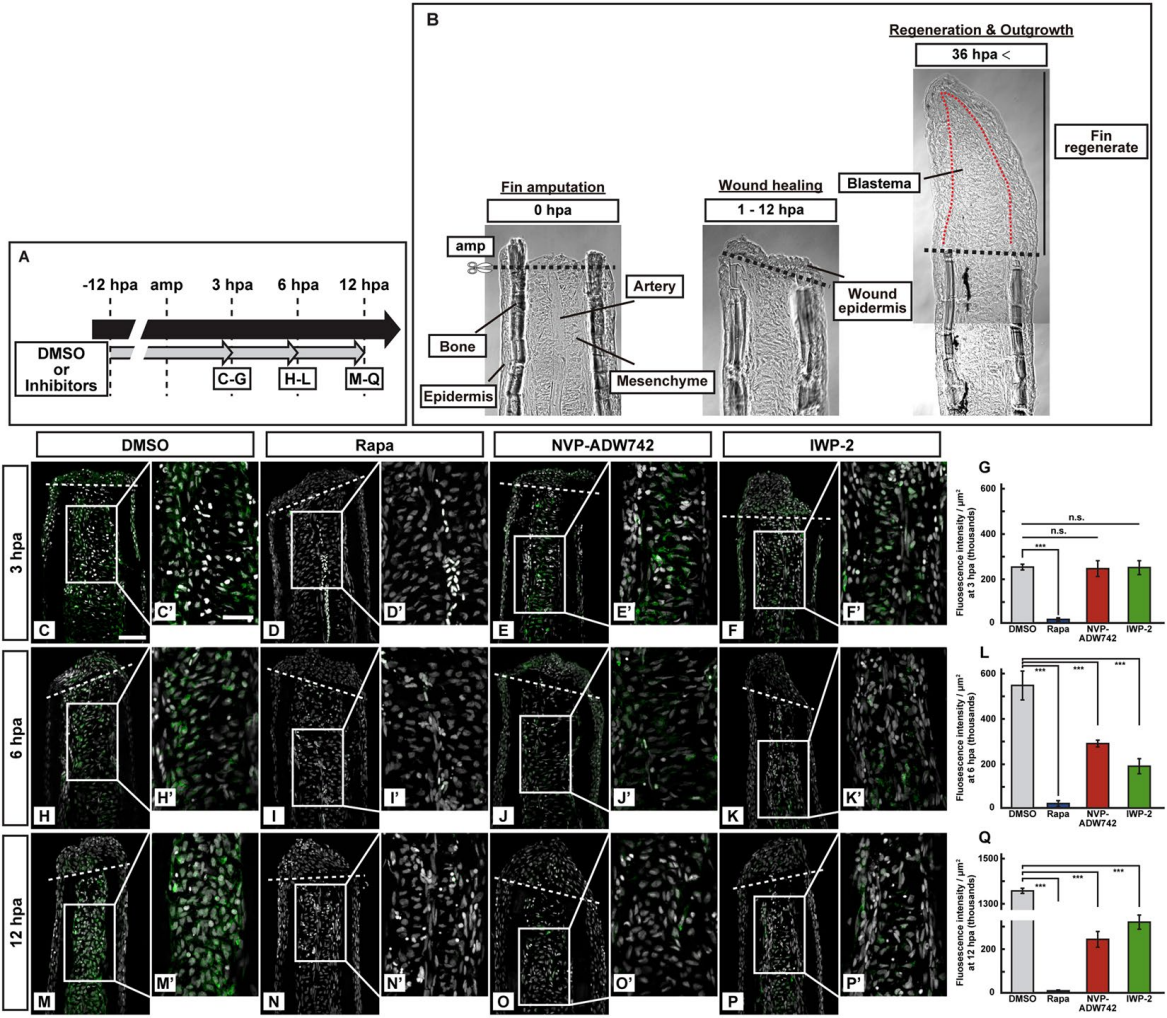
Activation of S6K was not inhibited by pharmacological inhibitors of IGF or Wnt signaling at 3 hpa. (**A**) Scheme of inhibitor treatments for rapamycin (a mTORC1 inhibitor), NVP-ADW742 (an IGF-1R inhibitor), and IWP-2 (a Wnt/β-catenin inhibitor) from −12 to 12 hpa. (**B**) Bright-field images of longitudinal ray sections of wild-type (WT) stumps (0 and 1–12 hpa) and fin regenerates (36 hpa). Black and red dotted lines indicate the amputation planes (amp) and the border between epidermis and blastema, respectively. (**C**–**F’**,**H**–**K’**, and **M**–**P’**) Longitudinal ray sections of DMSO- or inhibitors-treated WT fin stumps that were immunohistochemically stained with an antibody against p-S6K (green) at 3, 6, and 12 hpa (n = 5). 4,6-diamidino-2-phenylindole dihydrochloride (DAPI) fluorescent signal (grayscale; pseudo color) indicates the presence of nuclei. Representative images used for quantification are shown in (**G**,**L**, and **Q**) along with a highly magnified view. White dashed lines indicate the amputation planes. Scale bars: 50 μm (**C**–**F**,**H**–**K**, and **M**–**P**) and 25 μm (**C’**–**F’**,**H’**–**K’**, and **M’**–**P’**). (**G**,**L**, and **Q**) Quantification of the p-S6K fluorescence intensities per area that consist of the whole regenerates and 500 μm below the amputation plane (including the intra-ray, epidermal, and wound epidermal cells) in rapamycin-, NVP-ADW742-, or IWP-2-treated fin stumps at 3 (**G**), 6 (**L**), and 12 (**Q**) hpa (n = 5). n.s.: not significant. ***p < 0.001 by Student’s *t* test. Error bars represent the standard error.

We have previously reported that mTORC1 activation was regulated by both IGF and Wnt signaling pathways at 24 and 48 h post amputation (hpa) during zebrafish fin regeneration by using pharmacological inhibitors, NVP-ADW742 (an IGF-1R inhibitor) and IWP-2 (a Wnt/β-catenin inhibitor)^17^. However, amino acids and environmental stresses have been known to function as the upstream regulators of mTORC1 in addition to growth factors^14,15^. To explore the involvement of the upstream regulators other than growth factors in mTORC1 regulation, we carefully observed the expression of phosphorylated S6 kinase (p-S6K; activated form of S6K), a downstream target of mTORC1, during fin regeneration while IGF or Wnt signaling was inhibited. The p-S6K fluorescence intensities per area that consist of the whole regenerates and 500 μm below the amputation plane were significantly reduced via rapamaycin (rapa) treatment (Fig. 1C,D’, G–I’, L–N’, and Q). On the other hand, the p-S6K fluorescence intensities in IGF or Wnt signaling inhibited-fin stumps remained unchanged at 3 hpa (Fig. 1C,C’, and E–G), but were significantly reduced at 6 and 12 hpa when compared to those in the DMSO-treated fin stumps (Fig. 1H,H’, J–L, M,M’, and O–Q). The results of p-S6K fluorescence intensities at 3 and 12 hpa observed by immunohistochemical staining were further supported by western blotting analysis (Fig. S1), indicating that mTORC1 activation is not under the control of IGF and Wnt signaling at 3 hpa during fin regeneration.

### Lysosomal acidification, possibly through v-ATPase, is required for mTORC1 activation

**Figure 2:**
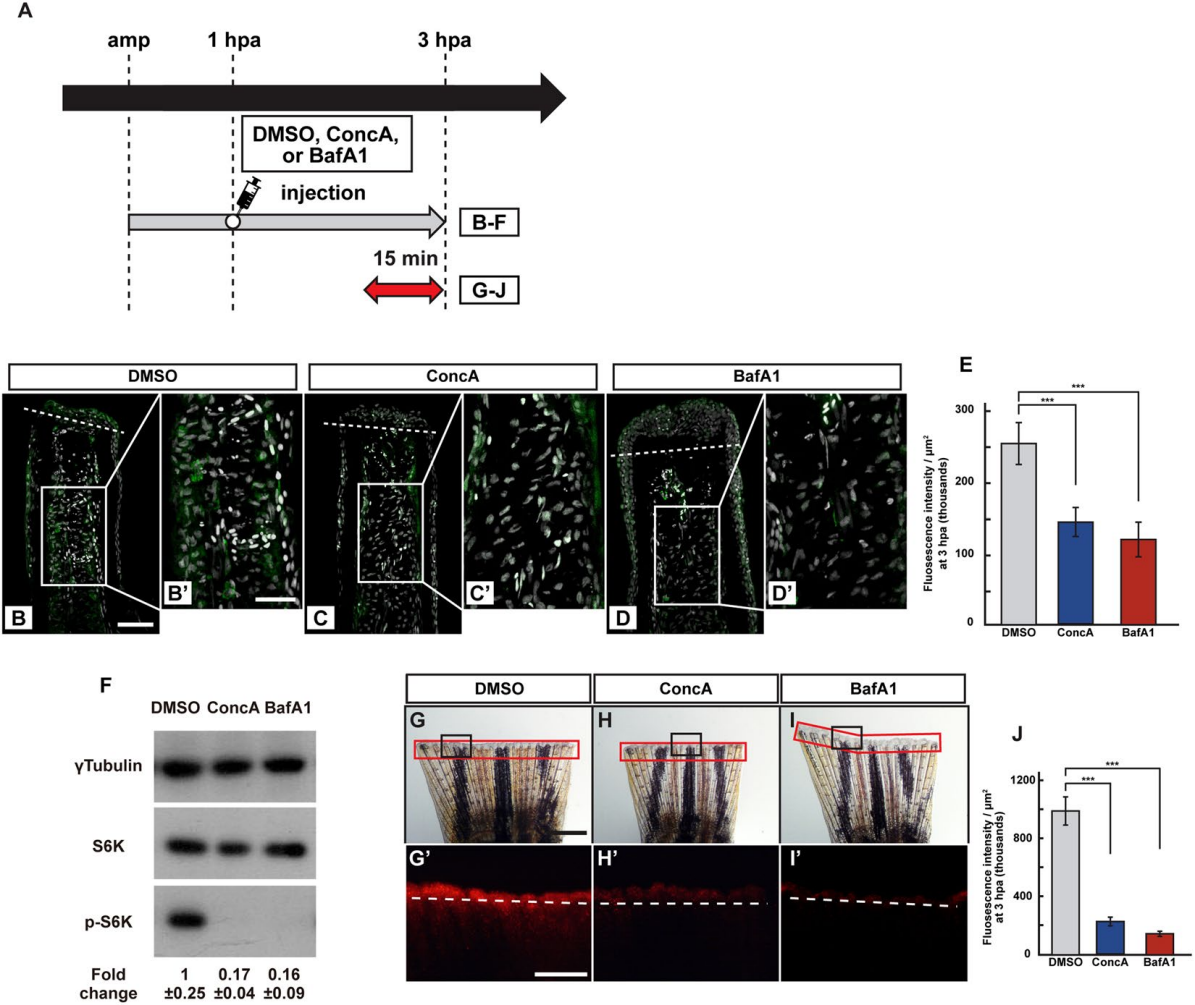
v-ATPase activity is required for the S6K activation. (**A**) Experimental scheme. DMSO, ConcA, or BafA1 solution was injected into the amputated fins at 1 hpa. The red two-headed-arrow indicates LysoTracker treatment, which was applied 15 min before observation. (**B**–**E**) Longitudinal ray sections and quantification of p-S6K fluorescence intensities per area that consist of the whole regenerates and 500 μm below the amputation plane in DMSO-, ConcA-, or BafA1-treated fin stumps at 3 hpa; p-S6K and nuclei were visualized by immunohistochemical staining and DAPI staining, respectively (n = 5). Representative images (**B**–**D’**) used for quantification are shown in (**E**). White dashed lines indicate the amputation planes. Scale bars: 50 μm (**B**–**D**) and 25 μm (**B’**–**D’**). ***p < 0.001 by Student’s *t* test. Error bars represent the standard error. (**F**) Western blotting analysis of γTubulin, S6K, and p-S6K in the DMSO-, ConcA-, or BafA1-treated fin stumps at 3 hpa (n = 6). γTubulin serves as a loading control. Numbers below each lane show the level of p-S6K in ConcA-, or BafA1-treated fin stumps relative to that in DMSO-treated fin stumps at 3 hpa normalized to loading control. (**G**–**I’**) Images of bright-field and fluorescence (red) microscopy, and quantification of the LysoTracker fluorescence intensity (see Materials and Methods) of DMSO-, ConcA-, or BafA1-treated fin stumps (n = 8). Black boxed areas in (**G**–**I**) are enlarged in (**G’**–**I’**), respectively. The LysoTracker fluorescence intensities in red boxed areas were measured (**G**–**I’**). Representative images (**G’**–**I’**) used for quantification are shown in (**J**). White dashed lines indicate the amputation planes. Scale bars: 1 mm (**G**–**H**) and 500 μm (**G’**–**H’**). ***p < 0.001 by Student’s *t* test. Error bars represent the standard error.

Previous studies using cell culture systems reported that the mTORC1 activation was closely linked to the SLC38A9/Rag/Regulator/v-ATPase complex on the lysosomal surface^13–15^. Interestingly, one of components in the lysosomal protein complex, v-ATPase, is required for zebrafish fin regeneration^16^. Therefore, we first examined the activity of the proton transporter, v-ATPase, during fin regeneration with the help of the LysoTracker, which is a fluorescent dye for labelling acidic lysosomes. High fluorescent signals of the LysoTracker were observed at 3 hpa at the amputation plane, which gradually decreased until 12 hpa (Fig. S2), showing the acidification of lysosomes during fin regeneration. To examine the relationship between the activation of v-ATPase and mTORC1, we further examined the functional inhibition of v-ATPase by using two different pharmacological inhibitors, Concanamycin A (ConcA)^18^ and Bafilomycin A1 (BafA1)^19^. We found that the p-S6K fluorescence intensity and p-S6K protein level were significantly reduced in each inhibitor-treated fin stump at 3 hpa by immunohistochemistry and western blotting, respectively (Fig. 2B–F). Lysosomal acidification at the amputation plane was suppressed by ConcA or BafA1 treatment at 3 hpa (Fig. 2G–J), suggesting that one of the mTORC1 regulators is a v-ATPase that regulates lysosomal acidification at this regeneration stage.

**Figure 3:**
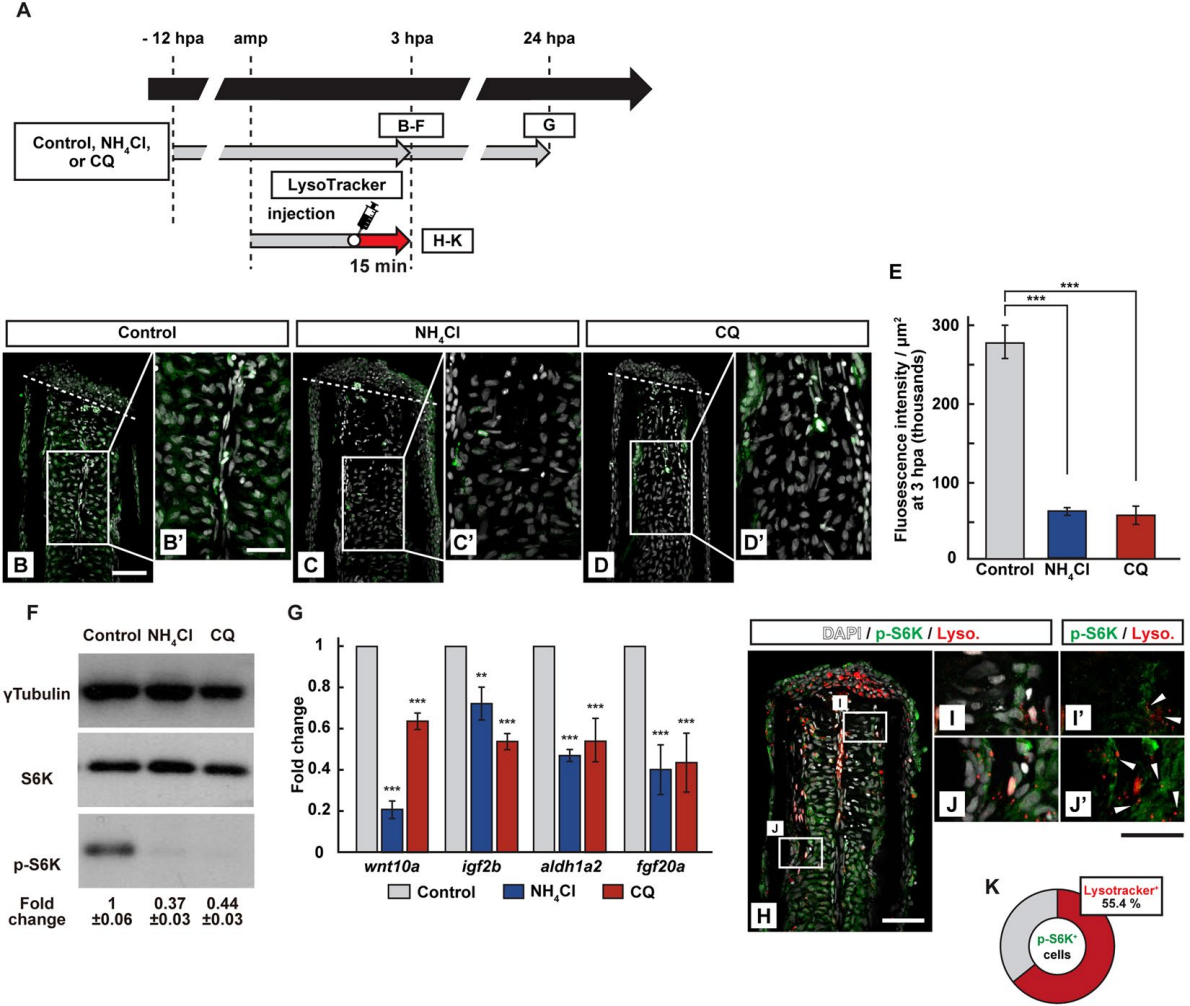
Requirement of lysosomal acidification in the S6K activation. (**A**) Experimental scheme. NH_4_Cl or chloroquine (CQ) was treated from −12 to 3 or 24 hpa (**B–G**). LysoTracker solution was injected into the amputated fins 15 min before fixation (**H–K**). (**B–E**) Longitudinal ray sections and quantification of p-S6K fluorescence intensities per area that consist of the whole regenerates and 500 μm below the amputation plane in control, NH_4_Cl-, or CQ-treated fin stumps at 3 hpa; p-S6K and nuclei were visualized by immunohistochemical staining and DAPI staining, respectively (n = 5). Representative images (**B**–**D’**) used for quantification are shown in (**E**). White dashed lines indicate the amputation planes. Scale bars: 50 μm (**B–D**) and 25 μm (**B’**–**D’**). ***p < 0.001 by Student’s *t* test. Error bars represent the standard error. (**F**) Western blotting analysis of γTubulin, S6K, and p-S6K in the DMSO-, NH_4_Cl-, or CQ-treated fin stumps at 3 hpa. γTubulin serves as a loading control. Numbers below each lane show the level of p-S6K in NH_4_Cl-, or CQ-treated fin stumps relative to that in DMSO-treated fin stumps at 3 hpa normalized to loading control. (**G**) The relative expression of growth factor-related genes in NH_4_Cl- or CQ-treated fin stumps by qPCR at 24 hpa. ***p < 0.001, **p < 0.05 by Student’s *t* test. Error bars represent the standard error. (**H**–**K**) Longitudinal ray sections and quantification of p-S6K^+^ and LysoTracker fluorescence^+^ cells in area that consist of the whole regenerates and 500 μm below the amputation plane at 3 hpa; p-S6K (green), nuclei (grayscale), and lysosomal acidification (red) were visualized by immunohistochemical staining, DAPI, and LysoTracker (Lyso.), respectively (n = 8). White boxed areas in (**H**) are enlarged in (**I**–**J’**), respectively. Representative images (**H**–**J’**) used for quantification are shown in (**K**). Arrowheads in (**I’** and **J’**) indicate LysoTracker fluorescence and p-S6K double positive cells. Scale bars: 50 μm (**H**) and 25 μm (**I**–**J’**). A pie chart shows that 55.4% of p-S6K^+^ cells are the LysoTracker fluorescence positive cells (**K**).

To evaluate the lysosomal acidification on mTORC1 activation, we next examined the inhibition of lysosomal acidification via treatment of ammonium chloride (NH_4_Cl) or chloroquine (CQ), both of which are the weak bases and function to increase the lysosomal pH^20^. The reagent treatment lead to a significant reduction of the p-S6K fluorescence intensity and p-S6K protein level at 3 hpa by immunohistochemistry and western blotting, respectively (Fig. 3B–F). We confirmed that lysosomal acidification was blocked in NH_4_Cl- or CQ-treated fin stumps at 3 hpa using LysoTracker (Fig. S3) and apoptosis was not increased by the reagent treatment at 3 and 24 hpa (Fig. S4). The expression of growth factor-related genes (*wnt10a*, *igf2b*, *aldh1a2*, and *fgf20a*)^11,12^ was significantly downregulated at 24 hpa in NH_4_Cl- or CQ-treated fin stumps (Fig. 3G). To further investigate the relationship between p-S6K and lysosomal acidification, we double stained fin stumps using the LysoTracker and p-S6K antibody. Approximately 55% of the p-S6K-positive cells were LysoTracker-positive in the fin stumps at 3 hpa (Fig. 3H–K), suggesting an association between mTORC1 activation and lysosomal acidification. Collectively, these results showed that lysosomal acidification is required to activate mTORC1 possibly via v-ATPase activity.

### Lysosomal acidification and mTORC1 activation are dependent on the amputation plane along the P-D axis

**Figure 4:**
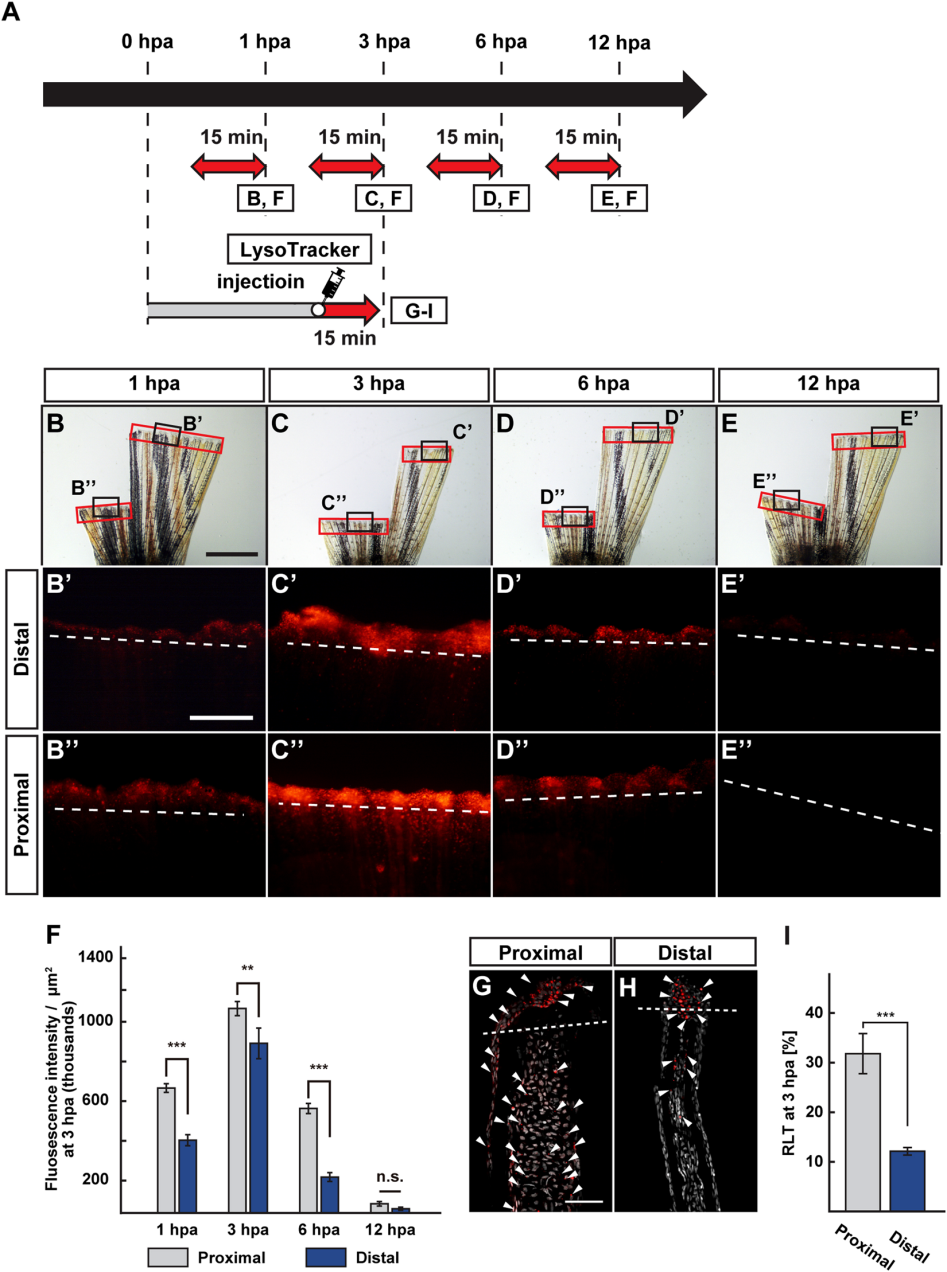
Position-dependent lysosomal acidification during fin regeneration. (**A**) Experimental scheme. Red two-headed-arrows indicate LysoTracker treatment, which was applied 15 min before observation. For cryosections, LysoTracker solution was injected into the amputated fins 15 min before fixation. (**B**–**E”**) Bright-field and fluorescent images of LysoTracker-treated fins at 1, 3, 6, and 12 hpa (n = 5). Black boxed areas in (**B**–**E**) are enlarged in (**B’**–**E’** and **B”**–**E”**), respectively. The LysoTracker fluorescence intensities in red boxed areas were measured (**B**–**E”**). Representative images (**B’–E”**) used for quantification are shown in (**F**). White dashed lines indicate the amputation planes. Scale bars: 3 mm (**B**–**E**) and 500 μm (**B’**–**E’** and **B”**–**E”**). (**F**) Quantification of Lysotracker fluorescence intensities at the proximal and distal positions at 1, 3, 6, and 12 hpa (n = 5). n.s.: not significant. ***p < 0.001, **p < 0.05 by Student’s *t* test. Error bars represent the standard error. (**G**–**I**) Longitudinal ray sections and quantification of ratios of LysoTracker fluorescence-positive cell number/total cell number (RLTs) within 500 μm of the amputation plane at 3 hpa (n = 5). Representative images (**G**,**H**) used for quantification are shown in (**I**). Arrowheads indicate LysoTracker fluorescence-positive cells (red). Scale bars: 50 µm (**G**,**H**) n.s.: not significant. ***p < 0.001 by Student’s *t* test. Error bars represent the standard error.

**Figure 5:**
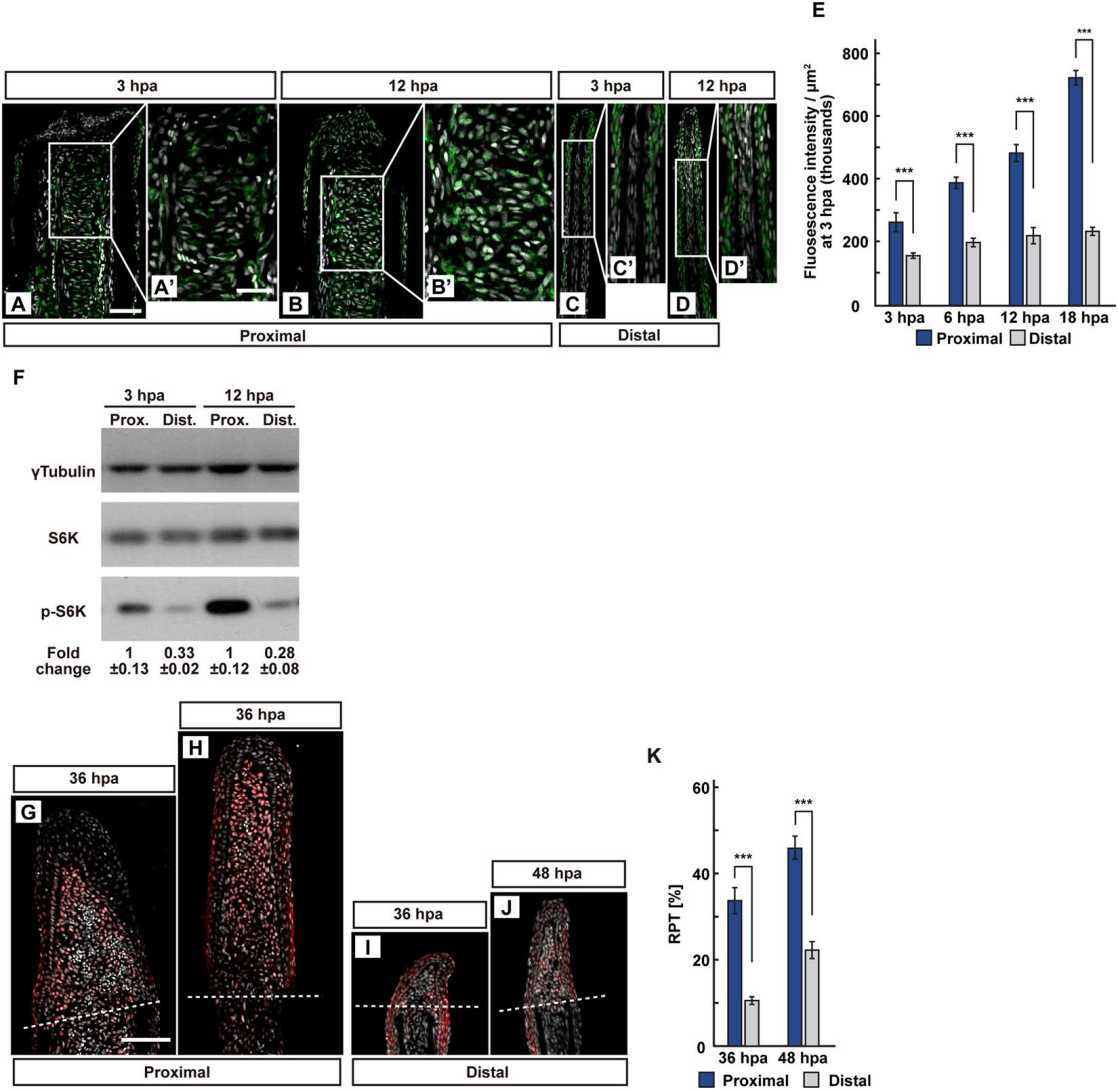
The position-dependent activation of S6K during fin regeneration. (**A**–**E**) Longitudinal ray sections (3 and 12 hpa) and quantification of p-S6K fluorescence intensities per area that consist of the whole regenerates and 500 μm below the amputation plane (3, 6, 12, and 18 hpa) of proximal and distal positions in WT fins; p-S6K and nuclei were visualized by immunohistochemical staining and DAPI staining, respectively (n = 8). Representative images (**A**–**D’**) used for quantification are shown in (**E**). White dashed lines indicate the amputation planes. Scale bars: 50 μm (**A**–**D**) and 25 μm (**A’**–**D’**). ***p < 0.001 by Student’s *t* test. Error bars represent the standard error. (**F**) Western blotting analysis of γTubulin, S6K, and p-S6K in WT fin stumps at 3 and 12 hpa. γTubulin serves as a loading control. Numbers below each lane show the level of p-S6K in proximal fin stumps relative to that in distal fin stumps at 3 or 12 hpa normalized to loading control, respectively. (**G**–**J**) Longitudinal ray sections of proximal and distal fins in WT fins at 36 and 48 hpa; PCNA and nuclei were visualized by immunohistochemical staining and DAPI staining, respectively (n = 5). Representative images (**G**–**J**) used for quantification are shown in (**K**). White dashed lines indicate the amputation planes. Scale bars: 50 μm (**G**–**J**). (**K**) Ratios of PCNA-positive cell number/total cell number (RPTs) in whole regenerates at 36 and 48 hpa (n = 5). ***p < 0.001 by Student’s *t* test. Error bars represent the standard error.

A previous report showed that the expression of *atp6v1e1b*, a v-ATPase family gene, and the H^+^ efflux in the proximal regenerates were higher than those in the distal regenerates at 12 hpa^16^. These results prompted us to analyze whether the acidification of lysosome was dependent on the position of the amputation plane along the P-D axis. LysoTracker fluorescence was detected at both proximal and distal amputation planes from 1 hpa (Fig. 4B–E”). The fluorescence intensities at the proximal regions were significantly higher than those at the distal regions, and this difference in fluorescence intensities remained until 6 hpa (Fig. 4F). Cryosections under the confocal microscope showed that the fluorescent LysoTracker-positive cells were observed in the epidermis and fin rays of proximal and distal fin stumps at 3 hpa (Fig. 4G,H). Moreover, the ratio of the fluorescent LysoTracker-positive cell number/total cell number (RLT) within the area that consist of the whole regenerates and 500 μm below the amputation plane in proximal stumps was significantly higher than that in distal stumps at 3 hpa (Fig. 4I). Because lysosomal acidification is required for mTORC1 activation, the position-dependence of p-S6K- and proliferating cell nuclear antigen (PCNA, a maker for proliferative cells^21^)-positive cells was also examined during fin regeneration. We found that p-S6K fluorescence intensity and p-S6K protein level in the proximal regions were significantly higher than those in the distal regions during fin regeneration at 3 and 12 hpa by immunohistochemistry and western blotting, respectively (Fig. 5A–F). Consistent with previous results that reported the regulation of cell proliferation via mTORC1^17^, the ratios of PCNA-positive cell number/total cell number (RPTs) within the area that consist of the whole regenerates also showed dependence on the amputation plane at 36 and 48 hpa (Fig. 5G–K). Our results suggest that position-dependent lysosomal acidification may cause the differences in mTORC1 activation between the proximal and distal amputation planes.

### L-type amino acid transporter 1 is required for mTORC1 activation

**Figure 6:**
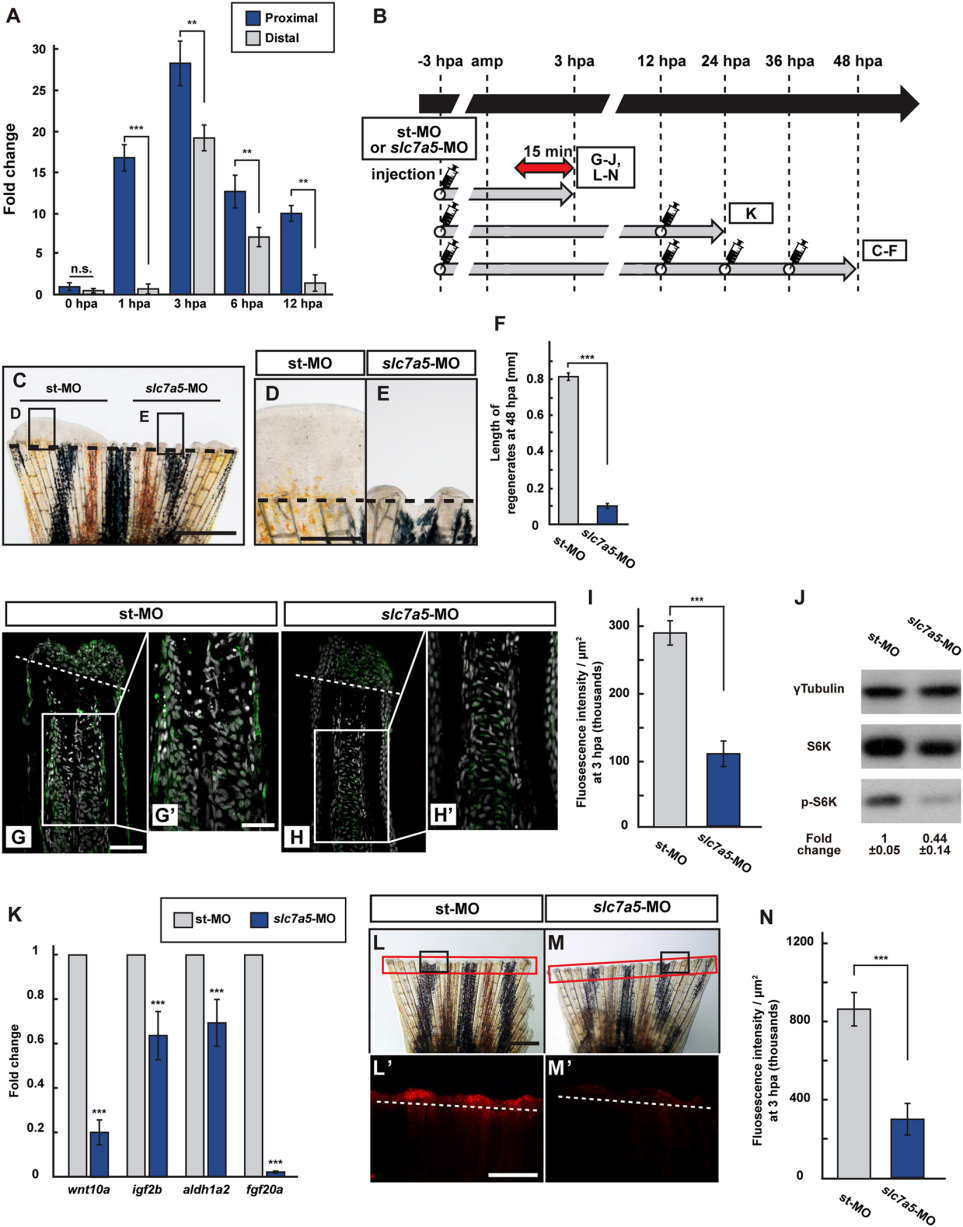
Slc7a5 is required for S6K activation and lysosomal acidification. (**A**) The relative expression of *slc7a5* in proximal and distal fin regenerates at 0, 1, 3, 6, and 12 hpa by qPCR analyses. (**B**) Scheme of vivo-MO mediated knockdown experiments from −3 to 48 hpa. A red two-headed-arrow indicates LysoTracker treatment, which was applied 15 min before observation. Syringes indicate vivo-MO injection. (**C**–**F**) Outgrowth of fin regenerates and quantification of their length after being injected with standard vivo-MO (st-MO) or *slc7a5* vivo-MO (*slc7a5*-MO) at 48 hpa (n = 5). The boxed areas in (**C**) are enlarged in (**D** and **E**), respectively. Representative images (**C**–**E**) used for quantification are shown in (**F**). Black dashed lines indicate the amputation planes. Scale bars: 1 mm (**C**) and 250 μm (**D**,**E**). ***p < 0.001 by Student’s *t* test. Error bars represent the standard error. (**G**–**I**) Longitudinal ray sections and quantification of p-S6K fluorescence intensities per area that consist of the whole regenerates and 500 μm below the amputation plane in st-MO or *slc7a5*-MO injected fin stumps at 3 hpa; p-S6K and nuclei were visualized by immunohistochemical staining and DAPI staining, respectively (n = 8). Representative images (**G**–**H’**) used for quantification are shown in (**I**). Scale bars: 50 μm (**G** and **H**) and 25 μm (**G’**–**H’**). ***p < 0.001 by Student’s *t* test. Error bars represent the standard error. (**J**) Western blotting analysis of γTubulin, S6K, and p-S6K in st-MO or *slc7a5*-MO injected fin stumps at 3 hpa (n = 5). γTubulin serves as a loading control. Numbers below each lane show the level of p-S6K in st-MO injected fin stumps relative to that in *slc7a5*-MO injected fin stumps at 3 hpa normalized to loading control. (**K**) The relative expression of growth factor-related genes in st-MO- or *slc7a5*-MO-injected fins by qPCR at 24 hpa. ***p < 0.001. Error bars represent the standard error. (**L**–**N**) Images of bright-field and fluorescence microscopy, and quantification of LysoTracker fluorescence intensities in st-MO- or *slc7a5*-MO-injected fin stumps at 3 hpa (n = 5). Black boxed areas in L and M are enlarged in L’ and M’, respectively. The LysoTracker fluorescence intensities in red boxed areas were measured (**L**–**M’**). Representative images (**L’**–**M’**) used for quantification are shown in (**N**). Scale bars: 1 mm (**L** and **M**) and 500 μm (**L’** and **M’**). ***p < 0.001. Error bars represent the standard error.

Previous studies implicated branched-chain amino acids, especially leucine, and their L-type amino acid transporters (LATs) as effective activators of mTORC1^22–25^. Because there are five LATs (LAT1; Slc7a5, LAT2; Slc7a8a and Slc7a8b, LAT3; Slc43a1a and Slc43a1b) in zebrafish as per the zebrafish database, we first examined the expression of these *LATs* at 0, 1, 3, 6, and 12 hpa. Among them, *slc7a5* showed the highest increase in expression levels at 3 hpa, and its expression in the proximal region was significantly higher than that in the distal region (Figs 6A and S5). Therefore, we further performed vivo-morpholino oligo nucleotide (MO)-mediated *slc7a5* knockdown experiments during fin regeneration. We first confirmed that a previously reported *slc7a5*-MO^26^ blocked the exon1 splicing of *slc7a5* pre-mRNA detected via quantitative real-time PCR (qPCR) (Fig. S6). Formation of blastema, a heterogeneous population of progenitor cells, was suppressed in *slc7a5*-MO-injected fin regenerates when compared to in standard MO (st-MO)-injected fin regenerates at 48 hpa (Fig. 6C–F). Consistent with the suppressed blastema formation, p-S6K fluorescence intensity, p-S6K protein level, and expression of growth factor-related genes were significantly reduced in *slc7a5*-MO-injected fins (Fig. 6G–K). We further found via LysoTracker analysis that lysosomal acidification was significantly reduced in *slc7a5*-MO-injected fin stumps when compared to in st-MO-injected fin stumps (Fig. 6L–N). In addition to knockdown experiments, we have used a Slc7a5-specific inhibitor, JPH203^27^, to block the cellular entry of amino acids. Similar to vivo-MO mediated knockdown experiments, blastema formation at 48 hpa, the p-S6K fluorescence intensity, p-S6K protein level, and lysosomal acidification by LysoTracker were significantly reduced at 3 hpa in JPH203-treated fin stumps when compared to those in DMSO-treated fins (Fig. S7). These our findings suggest that amino acid signaling through the Slc7a5 transporter is involved in mTORC1 activation possibly via lysosomal acidification during fin regeneration.

### Treatment with leucine and glutamine increases mTORC1 signaling and cell proliferation in both proximal and distal fin stumps

**Figure 7:**
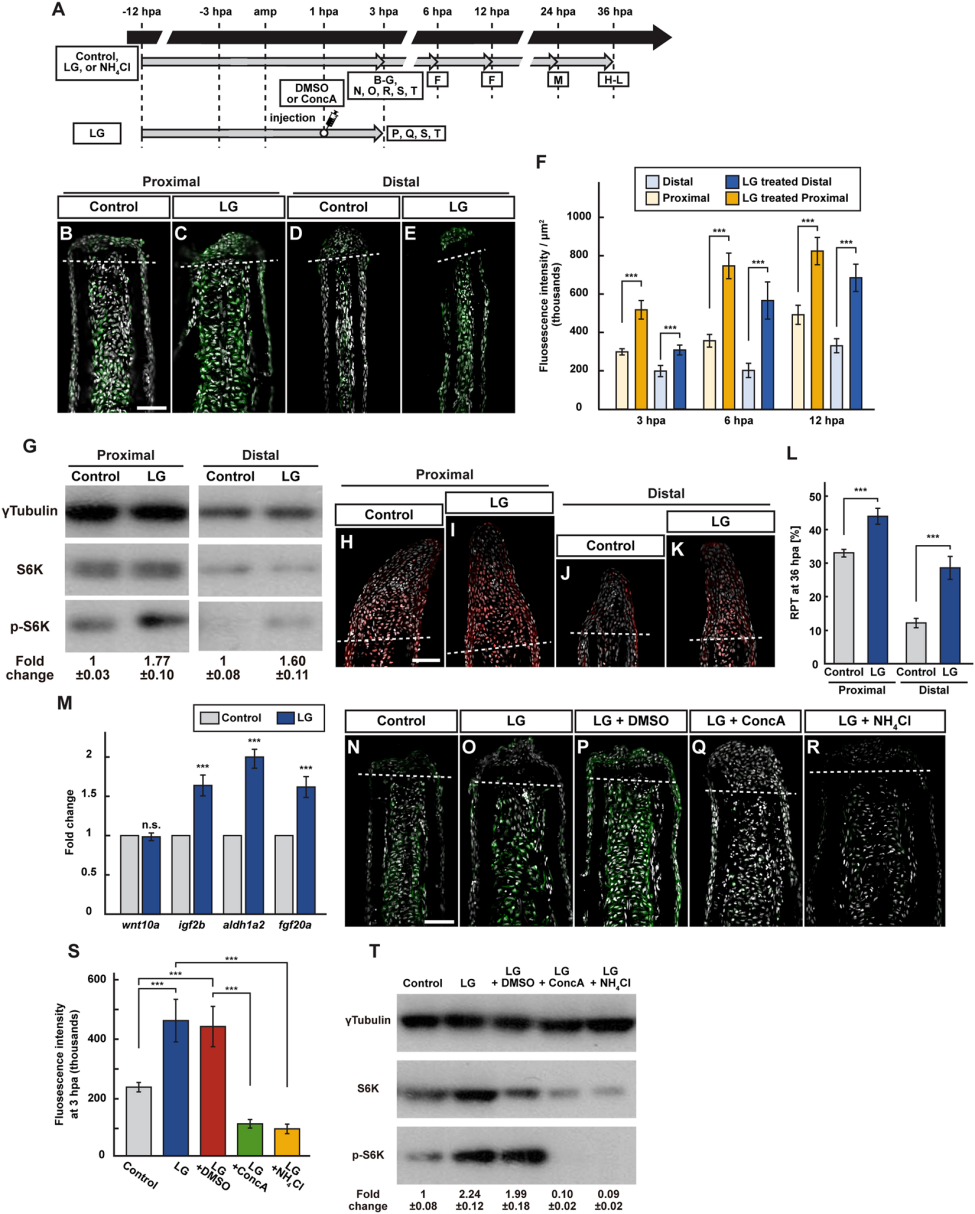
Leucine and glutamine treatment activates mTORC1 signaling via v-ATPase activity and lysosomal acidification. (**A**) Experimental scheme. (**B–F**) Longitudinal ray sections (3 hpa) and quantification of p-S6K fluorescence intensities per area that consist of the whole regenerates and 500 μm below the amputation plane (3, 6, and 12 hpa) of proximal and distal positions in control or LG-treated fin regenerates; p-S6K and nuclei were visualized by immunohistochemical staining and DAPI staining, respectively (n = 6). Representative images (**B–E**) used for quantification are shown in (**F**). White dashed lines indicate the amputation planes. Scale bars: 50 μm. ***p < 0.001 by Student’s *t* test. Error bars represent the standard error. (**G**) Western blotting analysis of γTubulin, S6K, and p-S6K in proximally and distally amputated LG-treated fin stumps at 3 hpa. γTubulin serves as a loading control. Numbers below each lane show the level of p-S6K in proximally or distally amputated LG-treated fin stumps relative to that in proximally or distally amputated control fin stumps at 3 hpa normalized to loading control, respectively. (**H–L**) Longitudinal ray sections and RPT quantification in proximally and distally amputated control or LG-treated fin regenerates at 36 hpa; PCNA and nuclei were visualized by immunohistochemical staining and DAPI staining, respectively (n = 5). Representative images (**H–K**) used for quantification are shown in (**L**). White dashed lines indicate the amputation planes. Scale bars: 50 μm. ***p < 0.001 by Student’s *t* test. Error bars represent the standard error. (**M**) The relative expression of growth factor-related genes in control and LG-treated fin regenerates by qPCR at 24 hpa. n.s.: not significant. ***p < 0.001 by Student’s *t* test. Error bars represent the standard error. (**N**–**S**) Longitudinal ray sections and quantification of p-S6K fluorescence intensities per area that consist of the whole regenerates and 500 μm below the amputation plane in control, LG-treated, LG-treated and DMSO-injected (LG + DMSO), LG-treated and ConcA-injected (LG + ConcA), or LG- and NH_4_Cl-treated (LG + NH_4_Cl) fin stumps at 3 hpa; p-S6K and nuclei were visualized by immunohistochemical staining and DAPI staining, respectively (n = 5). Representative images (**N**–**R**) used for quantification are shown in (**S**). White dashed lines indicate the amputation planes. Scale bars: 50 μm. ***p < 0.001 by Student’s *t* test. Error bars represent the standard error. (**T**) Western blotting analysis of γTubulin, S6K, and p-S6K in control, LG-treated, LG + DMSO, LG + ConcA, or LG + NH_4_Cl fin stumps at 3 hpa (n = 6). γTubulin serves as a loading control. Numbers below each lane show the level of p-S6K in LG-treated, LG + DMSO, LG + ConcA, or LG + NH_4_Cl fin stumps relative to that in control fin stumps at 3 hpa normalized to loading control.

A recent study showed that mTORC1 signaling stimulated by leucine and glutamine rescues defects associated with Roberts Syndrome in zebrafish^28^. Based on this finding, we wondered if the amino acids, leucine and glutamine, were involved in mTORC1 activation during fin regeneration. To explore this possibility, we carried out leucine and/or glutamine treatment in fin-amputated fish. Fin stumps after leucine or glutamine treatment showed no increased mTORC1 activation (Fig. S8), whereas co-treatment with leucine and glutamine (LG) significantly increased the p-S6K fluorescence intensity at 3, 6, 12 hpa and p-S6K protein level at 3 hpa in both proximal and distal fin stumps (Fig. 7B–G). In addition, although LysoTracker fluorescence intensities were not increased by leucine or glutamine treatment (Fig. S9B–K), they showed an LG concentration-dependent increase in LG-treated fin stumps compared to control fin stumps at 3 hpa (Fig. S9L–P). Consistent with increase of the p-S6K fluorescence intensities, RPTs in both proximal and distal regenerates were increased in LG-treated fin regenerates at 36 hpa (Fig. 7H–L). We further found that LG treatment significantly increased the expression of growth factor-related genes, except *wnt10a*, at 24 hpa (Fig. 7M). These results showed that LG signaling had the ability to change position-dependent cell proliferation via mTORC1 activation during fin regeneration. Because it is known that amino acid signaling is one of upstream regulators of mTORC1 via the SLC38A9/Rag/Regulator/v-ATPase complex^13–15^, we further tested the hierarchical relationship between LG signaling and v-ATPase/lysosomal acidification. The p-S6K fluorescence intensity and p-S6K protein level were significantly increased by LG treatments at 3 hpa; however, this increased level of mTORC1 signaling was suppressed by the co-treatment with ConcA or NH_4_Cl (Fig. 7N–T). Furthermore, up-regulation of LysoTracker fluorescence intensity by LG treatment was annulled by *slc7a5*-MO injection (Fig. S10). Results of the LG signal activation experiments, combined with the *slc7a5* knockdown data, suggest that LG and their transporter, Slc7a5, are upstream regulators of mTORC1 activity via v-ATPase/lysosomal acidification.

## Discussion

One of the most important processes in regeneration is the complete restoration of lost tissues or organs by cellular factors that regulate position-dependent cell proliferation and patterning (positional memory). Because the mTORC1 signaling pathway plays a central role in cell proliferation via the production of proteins, lipids, and nucleotides, and suppression of catabolic pathways such as autophagy, it is a potent candidate effector of position-dependent regeneration. In this study, we arrived at four conclusions. First, lysosomal acidification, possibly through v-ATPase activity, is required for mTORC1 activation. Second, both lysosomal acidification and mTORC1 activation are dependent on the amputation plane along the P-D axis. Third, an amino acid transporter Slc7a5 is necessary for mTORC1 activation and the expression of growth factor-related genes. Fourth, leucine and glutamine, which functions upstream of v-ATPase/lysosomal acidification, have the ability to increase cell proliferation via mTORC1 activation in both proximal and distal fin regenerates. Based on our results, we propose a molecular pathway that leads to the position-dependent cell proliferation during zebrafish caudal fin regeneration (Fig. S11). Leucine and glutamine are proximally enriched in unamputated fins as shown previously^10^. Leucine/glutamine signaling via Slc7a5 initiates v-ATPase activity in a position-dependent manner and causes lysosomal acidification. The position-dependent v-ATPase activity/lysosomal acidification activates the mTORC1 signaling, which leads to cell proliferation and upregulation of growth factor-related gene expression directly or indirectly.

In addition to leucine/glutamine, mTORC1 senses and responds to various stresses, such as energetic/metabolic stress and oxidative stress, but most of these stresses are repressive^29^. In contrast, a previous report using a cell culture system revealed that oxidative stress activates mTORC1 through modulating the TSC1/TSC2-Rheb GTPase pathway^30^. Oxidative stress is one of main regulation factors for zebrafish cauda fin regeneration: reactive oxygen species (ROS), such as H_2_O_2_, production was gradually increased at the amputation plane by 16 hpa^31^, and nerves and H_2_O_2_ levels controlled each other in a positive feedback loop^32^. Moreover, the spatial and temporal localization of ROS is closely similar to that of lysosome acidification. These previous data, combined with our results, suggest that oxidative stress might be one of upstream regulators for mTORC1 activation.

Double staining experiments with p-S6K and LysoTracker showed that approximately 55% of p-S6K-positive cells were fluorescent LysoTracker-negative at 3 hpa (Fig. 3H–K). A possible explanation for this result is that lysosomal acidification probably occurred before 3 hpa and therefore, LysoTracker fluorescence had already disappeared at 3 hpa. Another possibility is that upstream environmental signals of mTORC1 other than amino acids and growth factors, such as oxygen and energy, activate mTORC1 within 3 hpa. It would be pertinent to explore these upstream environmental signals that function in mTORC1 activation immediately after fin-amputation.

Recently, three amino acids (leucine, glutamine, and arginine) were implicated in the activation of mTORC1 through various amino acid sensing mechanisms^15,25,33^. Some amino acid sensing mechanisms for mTORC1 activation have been reported: binding of cytosolic leucine and Sestrin2, or cytosolic arginine and Cellular Arginine Sensor for mTORC1 (CASTOR1), prevents Sestrin2-GAP activity towards Rags2 (GATOR2), or CASTOR1-GATOR2 interaction via GATOR1 inactivation, leading to increased Rag-dependent mTORC1 signaling, respectively^15,25,33^; glutamine and leucine activate mTORC1 via glutaminolysis and α-ketoglutarate production upstream of Rag^34^; mTORC1 is activated by glutamine via adenosine diphosphate ribosylation factor-1 dependent on v-ATPase^35^. In this study, we showed that individual leucine or glutamine treatments had no effect on mTORC1 activation during fin regeneration, whereas treatment of both leucine and glutamine activated mTORC1 signaling. There are two possible explanations for this result; one is that both amino acids are required for mTORC1 activation as in the glutaminolysis and α-ketoglutarate production pathway, as mentioned above; while the other possibility is that, because Lat1 (Slc7a5) and 4F2hc (Slc3a2) are bi-directional transporters^36^, leucine is transported into the cytosol instead of the glutamine efflux. A recent study revealed that Lat1/4F2hc is recruited by lysosomal-associated transmembrane protein 4b (LAPTM4b) to the lysosome^37^. This recruitment leads to uptake of leucine into the lysosome and is required for mTORC1 activation depending on v-ATPase activity. These previous results are consistent with our results that show the axis of leucine/glutamine signaling-v-ATPase/lysosomal acidification is associated with mTORC1 activation. Further studies are needed to elucidate how leucine and glutamine activate mTORC1 during fin regeneration.

Previous reports revealed that the H^+^ pump v-ATPase activity is necessary for regeneration of *Xenopus* larval tail^38^ and zebrafish caudal fin^16^. In *Xenopus* larvae, v-ATPase mRNA and protein are expressed in the regeneration bud, and its protein is localized in the cell-membrane of the bud cells^38^. The v-ATPase activity changes membrane voltage in the bud cells via the H^+^ efflux and repolarization by its activity is necessary for tail outgrowth^38^. Like the *Xenopus* larval tail, H^+^ efflux via v-ATPase is required for blastema cell proliferation in zebrafish caudal fin regeneration at 24 and 48 hpa^16^. In addition to the previous study results, we also found that lysosomal acidification, possibly through v-ATPase, is required for activation of mTORC1 signaling within 3 hpa. To date, only one study reported that lysosomal pH functions not only in mTORC1 activation, but also in its deactivation via protein degradation in osteoclasts^39^. It is possible that lysosomal pH itself may affect the amino acid transportation into the lysosome, amino acid sensing in the lysosome, or formation of the Rag/Regulator/v-ATPase complex formation on the lysosome membrane. Our results combined with previous data showed that v-ATPase has dual functions in both lysosomal pH control and plasma membrane voltage for zebrafish caudal fin regeneration at different regeneration stages.

However, the most important finding of this study is that leucine/glutamine and v-ATPase/lysosomal acidification via mTORC1 activation are required for the position-dependent fin regeneration. A recent report showed by metabolomic analyses that many specific amino acids are proximally or distally enriched in uninjured zebrafish caudal fin along the P-D axis. Both leucine and glutamine were identified as proximally-enriched amino acids^10^. In addition, the expression of *atp6v1e1b*, a component of the v-ATPase complex, in the proximal regenerates was higher than that in the distal regenerates^16^. In this study, we also showed that the expression of *slc7a5*, encoding L-type amino acid transporter, in the proximal regenerates was higher than that in the distal regenerates at 3 hpa, and that treatment with leucine and glutamine lead to the upregulation of PCNA expression in the distal regenerates at 36 hpa. Furthermore, inhibition of leucine/glutamine signaling and lysosomal acidification resulted in downregulation of growth factor gene expression, such as *wnt10a*, *igf2b*, *aldh1a2*, and *fgf20a*. Cumulatively, these results suggest that the axis of leucine/glutamine signaling-v-ATPase/lysosomal acidification via mTORC1 activation is a potent candidate effector of the position-dependent regeneration along the P-D axis at the early stage of fin regeneration (within 3 hpa). Moreover, differences in cell proliferation along the P-D axis are regulated by mTORC1 or possibly by growth factors downstream of this axis.

## Materials and Methods

### Zebrafish husbandry, drug treatments, LysoTracker staining, amino acid treatments, and morpholino knockdown

All zebrafish experiments were conducted in strict accordance with relevant nation and international guidelines: ‘Act on Welfare and Management of Animals’ (Ministry of Environment of Japan), and were approved by the Hiroshima University Animal Research Committee (Permit Number: F17-2). For caudal fin amputation, adult zebrafish (AB/Tüebingen strain) were anesthetize using Tricaine (Sigma-Aldrich) and the caudal fins were amputated with razor blades along the dorsoventral axis. The proximal or distal amputations were performed as described previously^16^; for distal amputation, one or two ray segments were cut before the first ray bifurcation, and for proximal amputation, two segments distal to the most posterior scale covering the fin base were cut. In case amputation position is not specified, caudal fins were amputated at the proximal position.

Rapamycin (2.4 μM, LC Laboratories), NVP-ADW742 (5 μM, AdooQ Biosciences), IWP-2 (10 μM, Promega), CQ (4.5 mM, Sigma-Aldrich), NH_4_Cl (5 mM, Sigma-Aldrich), and JPH203 (40 μM, Chemescene) were used as specific inhibitors. All inhibitors, except CQ and NH_4_Cl, were dissolved in DMSO, with a 0.1% final DMSO concentration in fish water. The control fish were maintained in fish water (for CQ and NH_4_Cl) or with 0.1% DMSO (for rapamycin, NVP-ADW742, IWP-2, and JPH203) at 28.5 °C and this water containing respective drug was replaced daily. These treatments started 12 h before fin amputation. Two fish were placed in 50 ml of fish water containing respective drug and during the drug treatment, the fish were fed once a day. ConcA (200 nM, Santa Cruz Biotechnology) or BafA1 (200 nM, Cell Signaling) were dissolved in 100% DMSO and diluted in Danieau medium^16^. This diluted solution was injected into amputated fins at 1 hpa.

For LysoTracker staining, fin-amputated adult fish were incubated in LysoTracker Red DND-99 (ThermoFisher Scientific) solution diluted in fish water (1:1000) at 28.5 °C for 15 min before live imaging. To observe longitudinal ray sections, 2 nl of the diluted LysoTracker solution (1 μM) was injected directly into the 2^nd^, 3^rd^, and 4^th^ fin rays from the dorsal and ventral amputated fins below the amputation plane. For leucine and glutamine treatment, the fish were incubated in the fish water containing 5, 15, and 25 mM L-leucine (Sigma-Aldrich) and/or L-glutamine (Sigma-Aldrich). The fish water containing of the two amino acids was replaced every 12 h.

For MO knockdown experiments, we used vivo-MO targeted against *slc7a5* and standard control vivo-MO (st-MO) (GeneTools, LLC) as following: *slc7a5* vivo-MO (*slc7a5*-MO) 5′-AGGTAACAGTTTACTTACGTATACA-3′^26^, st-MO 5′-CCTCTTACCTCAGTTACAATTTTATA-3′. *slc7a5*-MO (1 mM) or st-MO (1 mM) was injected into each inter-ray 3 h before fin amputation, and then every 12 h before observation or harvest, as described previously^40^.

### Immunohistochemical staining and detection of cell death

The amputated fins were fixed overnight in 4% paraformaldehyde in 0.1 M phosphate-buffered saline at 4 °C, embedded in Tissue-Tek O.C.T compound (Sakura Finetek), and cryosectioned to 14 μm thickness by using a Leica CM3050S^17^. The following primary antibodies were used: anti-PCNA mouse monoclonal antibody at 1:1000 (Sigma, #P8825) and phospho-S6 ribosomal protein (Ser240/244) rabbit polyclonal antibody at 1:300 (Cell Signaling, #2215). The following secondary antibodies were used: Alexa Fluor^®^ 488 goat anti-rabbit IgG antibody at 1:500 (Invitrogen, Life Technologies Corp.) and Alexa Fluor^®^ 594 goat anti-mouse IgG antibody at 1:500 (Invitrogen, Life Technologies Corp.). 4′,6-diamidino-2-phenylindole (DAPI) was used for nuclei staining at a concentration of 1:1000. To detection apoptotic cells, we performed TUNEL staining using an *In situ* cell death detection kit (Roche, #11684795910) according to the manufacturer’s instructions. The images were captured using an Olympus FV1000-D confocal microscope with the same exposure times using the FluoView software. Eight optical sections per one cryosection along the z-axis were taken in 0.67 μm intervals, and the captured images were analyzed using ImageJ (NIH).

### qPCR analyses

Extraction of total RNA from regenerating fins was performed as described previously^41^. Five independent qPCR experiments of ten genes (*wnt10a*, *igf2b*, *aldh1a2*, *fgf20a*, *slc7a5*, *slc7a8a*, *slc7a8b*, *slc43a1a*, *slc43a1b*, and *ribosomal protein L13a* (*rpl13a*)) for gene expression and MO efficacy was performed in duplicates using the Thermal Cycler Dice Real-Time System II and SYBR Premix Ex Taq II (TAKARA) as described previously^42^. Zebrafish *rpl13a* was used as a reference gene. The qPCR primers are listed in Table S1.

### LysoTracker image analyses

Fluorescent LysoTracker images were acquired using a Leica MZ FLIII microscope and Penguin 600CL cooled CCD camera (Pixera). As described previously^43^, average LysoTracker fluorescence intensity was measured using the ImageJ software (NIH) with the following formula: [integrated intensity in the whole amputation plane area (W 500 μm × L full-length of the amputation plane) − mean fluorescence intensity of background].

### Western blotting

Western blotting was performed as described previously^44^. Fins were lysed in 2 × SDS-sample buffer (125 mM Tris-HCl (pH 6.8), 4% SDS, 20% Glycerol, 0.01% bromophenol blue, 2% 2-mercaptoethanol) containing a protease inhibitor cocktail (Roche, #11836153001) and a phosphatase inhibitor cocktail (Nacalai, #07574-61). Total proteins (5 µg) were separated by SDS-PAGE and transferred to a polyvinylidene fluoride (PVDF) membrane. The PVDF membranes were incubated overnight with primary antibodies: anti-γTubulin (Sigma-Aldrich, #T6557) at 1:20000, ribosomal protein S6 Antibody (C-8) (Santa Cruz Biotechnology, #sc-74459) at 1:1000, and anti-phospho-S6 ribosomal protein (Ser240/244) (Cell signaling, #2215) at 1:1000. For detection, horseradish peroxidase-coupled secondary antibodies: goat anti-mouse IgG-HRP at 1:10000 (Santa Cruz Biotechnology, #sc-2055) and mouse anti-rabbit IgG-HRP at 1:10000 (Santa Cruz Biotechnology, #sc-2357), and the ECL Prime Western Blotting Detection Reagent (GE Healthcare, #RPN2232) were used, according to the manufacturer’s instructions. A densitometric analysis of the western blotting was performed using ImageJ, with γTubulin for normalization.

### Statistical analyses

All experiments were performed independently more than three times and the results were reported as means ± Standard Error of Mean (SEM). More than 100 cells for distal amputation and 500 cells for proximal amputation were used for quantification of fluorescence intensity (for p-S6K and LysoTracker) or quantification of RLT, RPT, and percentage of TUNEL^+^ cells. Statistical significance was determined by using the Student’s *t*-test. *p* values of ≤0.05 were considered to be statistically significant.

## Electronic supplementary material

Supplementary Information
