# Supplementary material for: Leucine/glutamine and v-ATPase/lysosomal acidification via mTORC1 activation are required for position-dependent regeneration
Source: Sci Rep. 2018 May 29;8:8278. doi: 10.1038/s41598-018-26664-2 (PMC5974189; doi:10.1038/s41598-018-26664-2)
Supplement: Supplementary file 1 — Supplementary Information [file 41598_2018_26664_MOESM1_ESM.pdf]

Supplementary Information

Leucine/glutamine and v-ATPase/lysosomal acidification via mTORC1 activation are required for position-dependent regeneration

Kazuya Takayama, Akihiko Muto[#], and Yutaka Kikuchi[*]

Department of Biological Science, Graduate School of Science, Hiroshima University, Kagamiyama 1-3-1, Higashi-Hiroshima, Hiroshima, 739-8526 Japan

[*]Corresponding Author: Yutaka Kikuchi (E-mail yutaka@hiroshima-u.ac.jp)

[#]Present Affiliation: Hematology Business Development

HU Business Development, Sysmex Corporation

4-4-4 Takatsukadai, Nishi-ku, Kobe 651-2271, Japan

Keywords: position-dependent regeneration; mTORC1; lysosomal acidification; leucine; glutamine; zebrafish

Figure S1

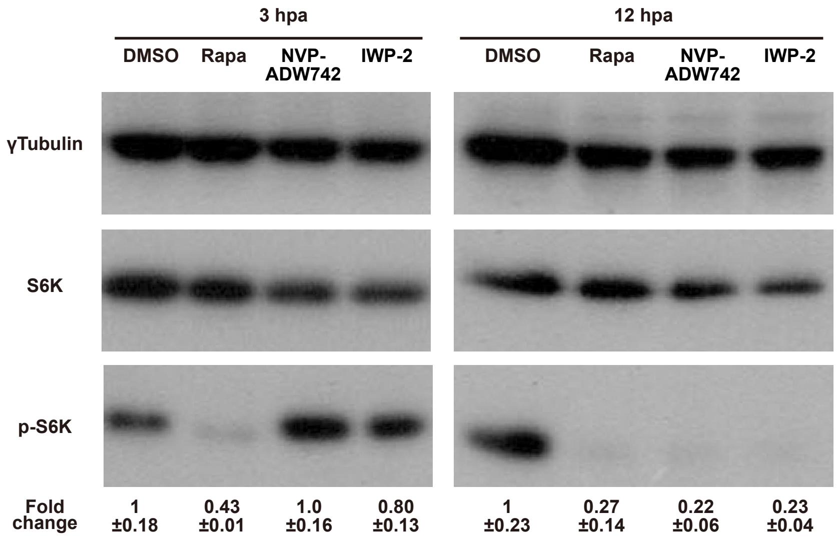
3 hpa
12 hpa
DMSO
Rapa
NVP-ADW742
IWP-2
DMSO
Rapa
NVP-ADW742
IWP-2
γTubulin
S6K
p-S6K
Fold change
1 ±0.18
0.43 ±0.01
1.0 ±0.16
0.80 ±0.13
1 ±0.23
0.27 ±0.14
0.22 ±0.06
0.23 ±0.04

Figure S1. Western blot analysis of rapamycin-, NVP-ADW742-, or IWP-2-treated fin stumps.

Western blot analysis of γTubulin, S6K, and p-S6K in rapamycin-, NVP-ADW742-, or IWP-2-treated fin stumps at 3 and 12 hpa (n = 5). γTubulin serves as a loading control. Numbers below each lane show the level of p-S6K in rapamycin-, NVP-ADW742-, or IWP-2-treated fin stumps relative to that in DMSO-treated fin stumps at 3 or 12 hpa normalized to loading control, respectively.

Figure S2

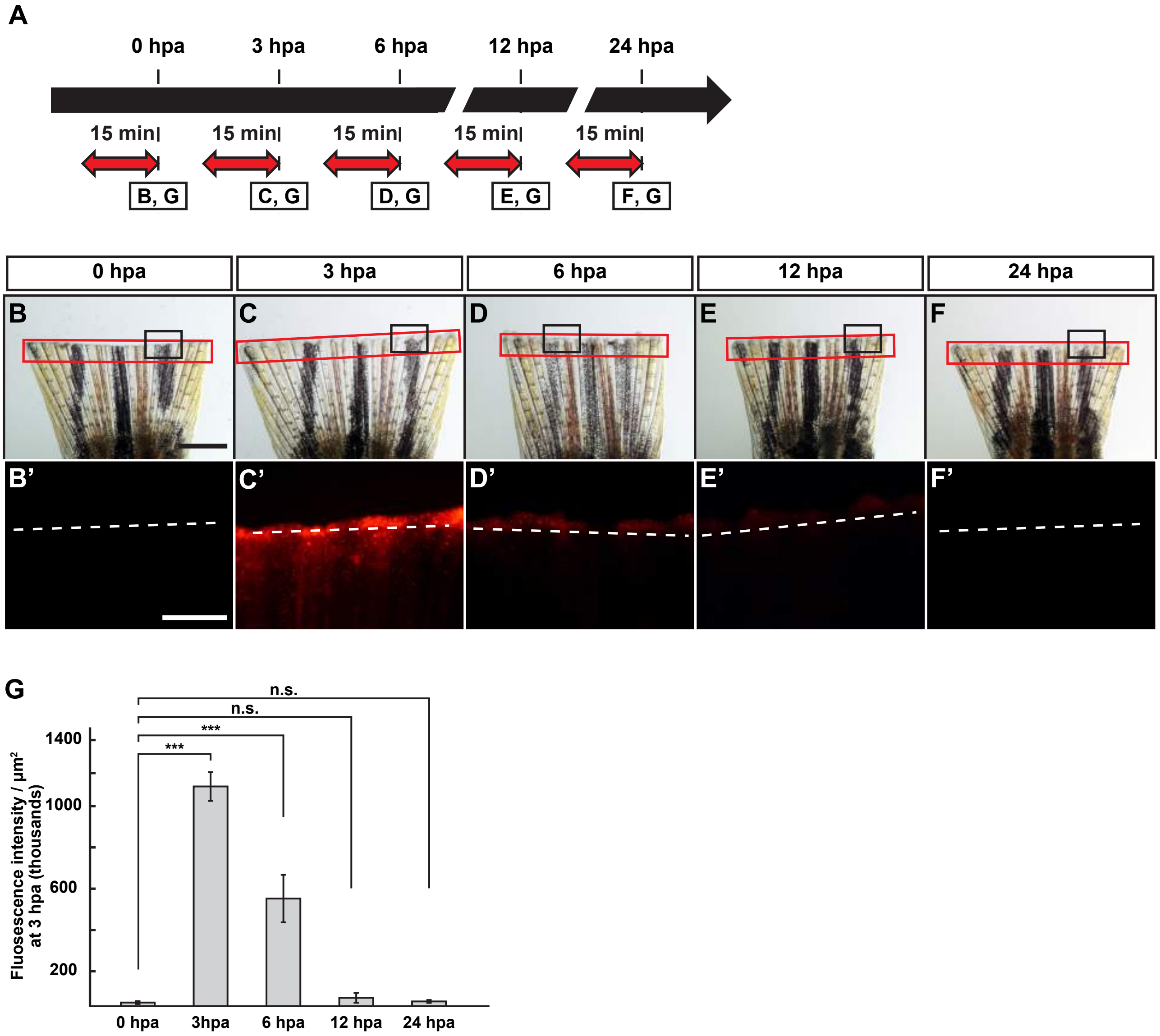
A
0 hpa
3 hpa
6 hpa
12 hpa
24 hpa
15 min
15 min
15 min
15 min
15 min
B, G
C, G
D, G
E, G
F, G
0 hpa
3 hpa
6 hpa
12 hpa
24 hpa
B
C
D
E
F
B'
C'
D'
E'
F'
G
n.s.
n.s.
***
***
Fluosescence intensity / µm²
at 3 hpa (thousands)
1400
1000
600
200
0 hpa
3hpa
6 hpa
12 hpa
24 hpa

Figure S2. Lysosomal acidification during fin regeneration.

(A) Experimental scheme. Red two-headed-arrows indicate LysoTracker treatment, which was applied 15 min before observation. (B-F') Images of bright-field and fluorescence microscopy, and quantification of LysoTracker fluorescence intensities at 0, 3, 6, 12, and 24 hpa (n = 5). Black boxed areas in B-F are enlarged in B'-F', respectively. The LysoTracker fluorescence intensities in red boxed areas were measured (B-F'). Representative images (B'-F') used for quantification are shown in G. White dashed lines indicate the amputation planes. Scale bars: 1 mm (B-F) and 500 μm (B'-F'). n.s.: not significant. ***$p < 0.001$ by Student's *t* test. Error bars represent the standard error.

Figure S3

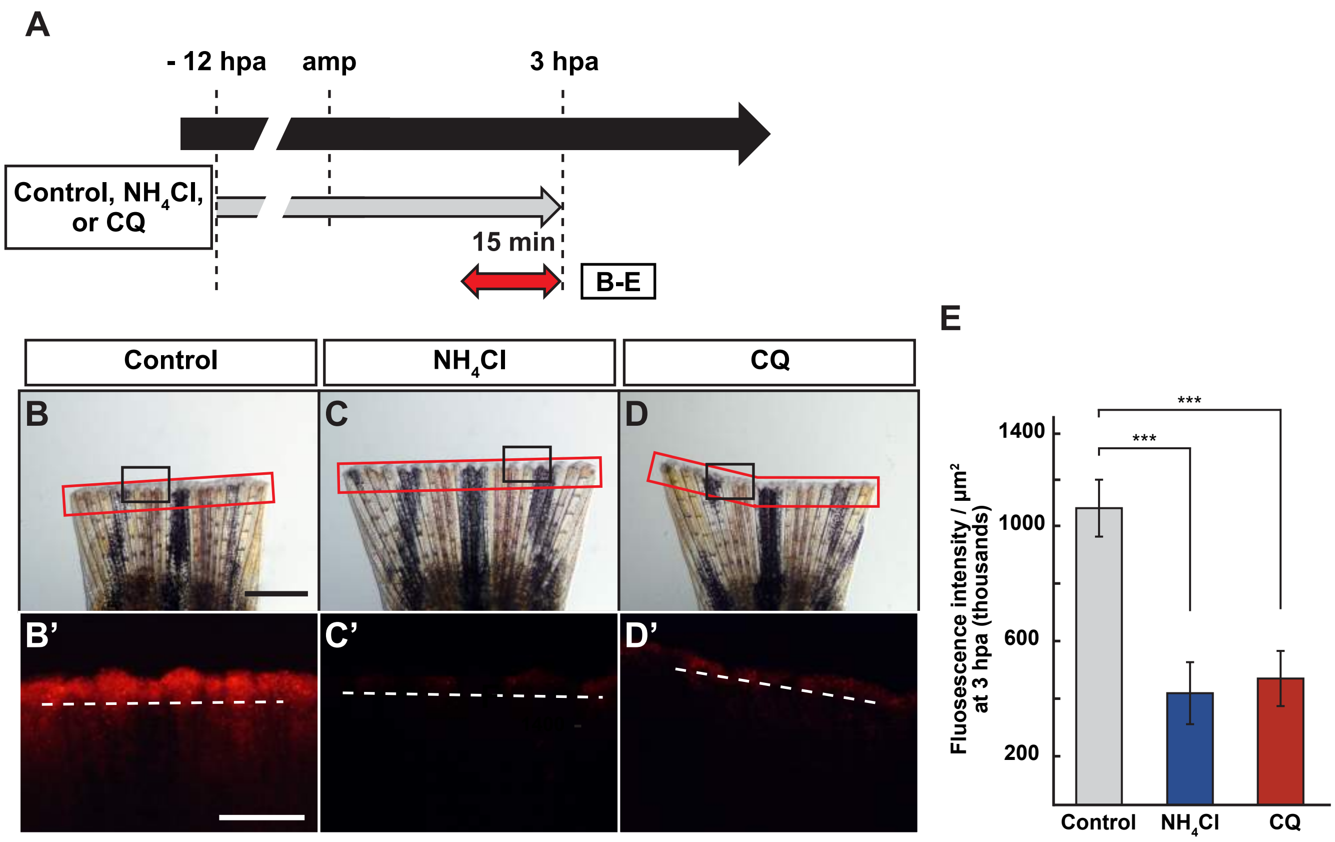
A
- 12 hpa
amp
3 hpa
Control, NH4Cl, or CQ
15 min
B-E
Control
NH4Cl
CQ
B
C
D
B'
C'
D'
E
***
***
1400
1000
600
200
Fluosescence intensity / μm2 at 3 hpa (thousands)
Control
NH4Cl
CQ

Figure S3. Lysosomal acidification in NH_4Cl- and CQ-treated fins.

(A) Experimental scheme. A red two-headed-arrows indicates LysoTracker treatment, which was applied 15 min before observation. (B-E) Images of bright-field and fluorescence microscopy, and quantification of LysoTracker fluorescence intensities in control, NH_4Cl-, or CQ-treated fins at 3 hpa (n = 8). Black boxed areas in B-D are enlarged in B'-D', respectively. The LysoTracker fluorescence intensities in red boxed areas were measured (B-D'). Representative images (B'-D') used for quantification are shown in E. White dashed lines indicate the amputation planes. Scale bars: 1 mm (B-D) and 500 μm (B'-D'). ***$p < 0.001$ by Student's t test. Error bars represent the standard error.

Figure S4

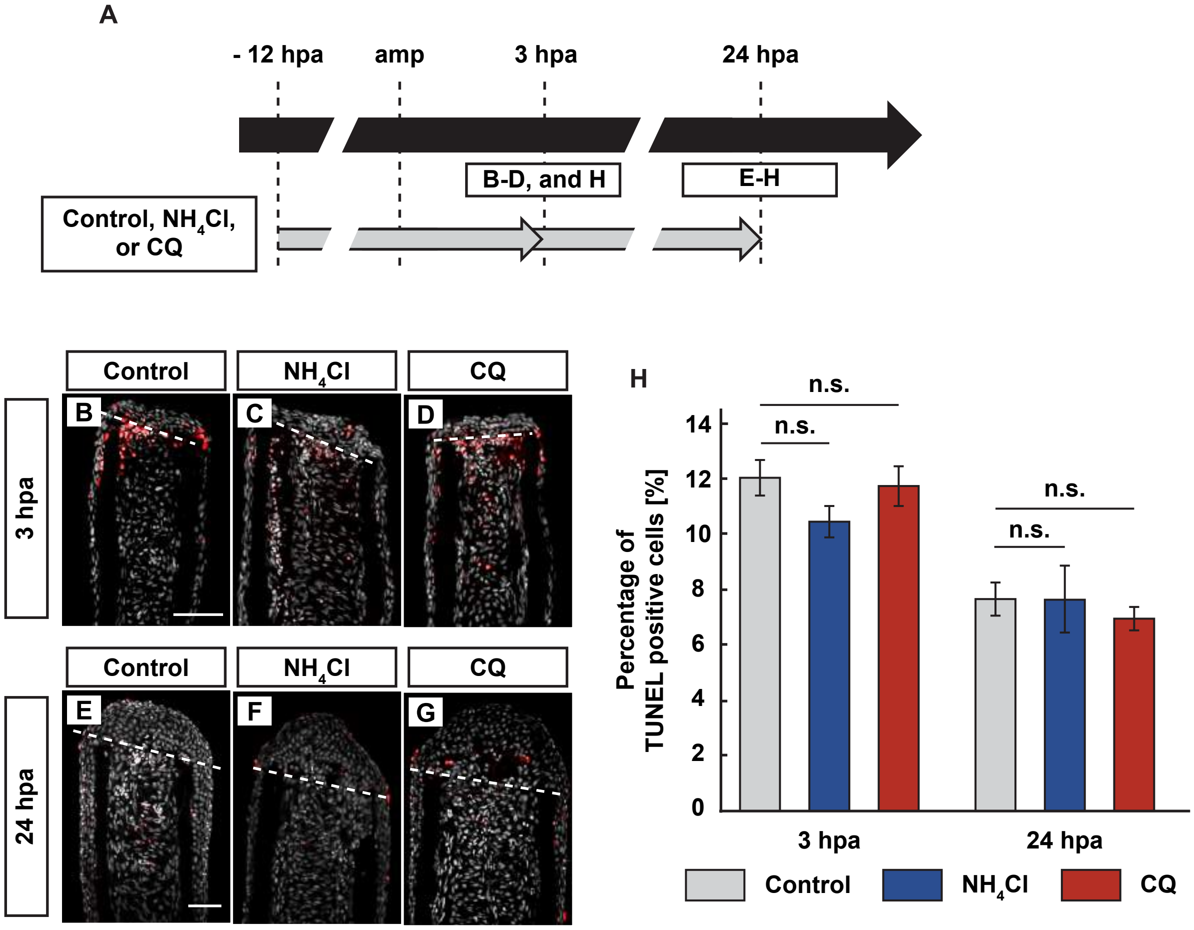

A
- 12 hpa
amp
3 hpa
24 hpa
B-D, and H
E-H
Control, NH4Cl, or CQ
Control
NH4Cl
CQ
3 hpa
B
C
D
24 hpa
E
F
G
H
n.s.
n.s.
n.s.
n.s.
Percentage of TUNEL positive cells [%]
0
2
4
6
8
10
12
14
3 hpa
24 hpa
Control
NH4Cl
CQ

Figure S4. Detection of apoptotic cells by NH_4Cl- or CQ-treatment.

(A) Experimental scheme. NH_4Cl or chloroquine (CQ) was treated from -12 to 3 or 24 hpa. (B-G) Longitudinal ray sections and quantification of TUNEL positive cells/total cell number per area that consists of the whole regenerates and 500 μm below the amputation plane in control, NH_4Cl-, or CQ-treated fin stumps at 3 and 24 hpa; apoptotic cells and nuclei were visualized by immunohistochemical staining and DAPI staining, respectively (n = 5). Representative images (B-G) used for quantification are shown in H. White dashed lines indicate the amputation planes. Scale bars: 50 μm (B-G). n.s.: not significant. Error bars represent the standard error.

Figure S5

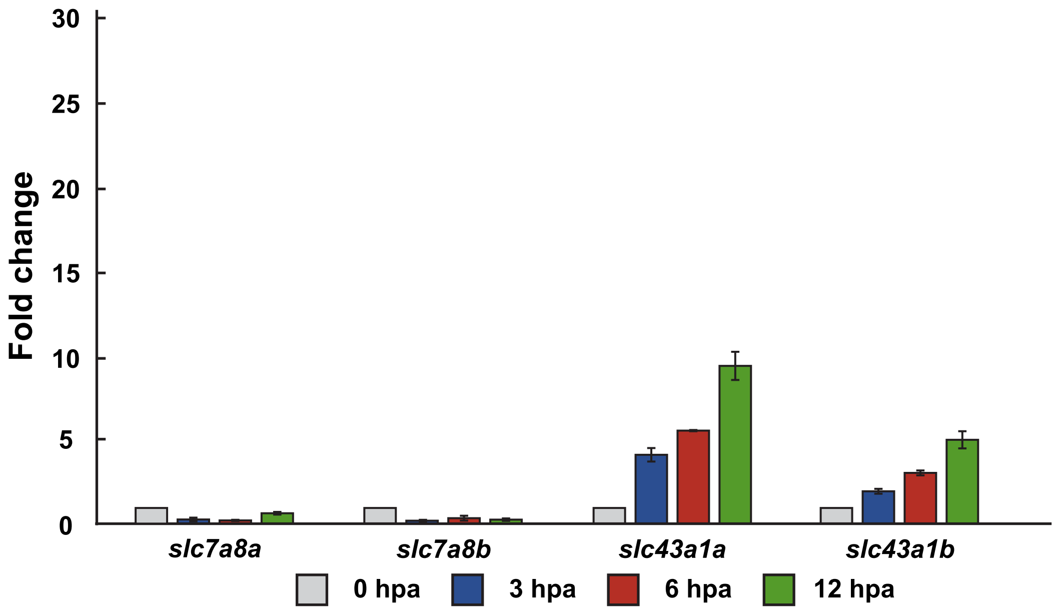

30
25
20
15
10
5
0
Fold change
slc7a8a
slc7a8b
slc43a1a
slc43a1b
0 hpa
3 hpa
6 hpa
12 hpa

Figure S5. Expression of *LATs* genes during fin regeneration.

The relative expression of the four *LATs* genes (*slc7a8a*, *slc7a8b*, *slc43a1a*, and *slc43a1b*) at 0, 3, 6, and 12 hpa by qPCR. Error bars represent the standard error.

Figure S6

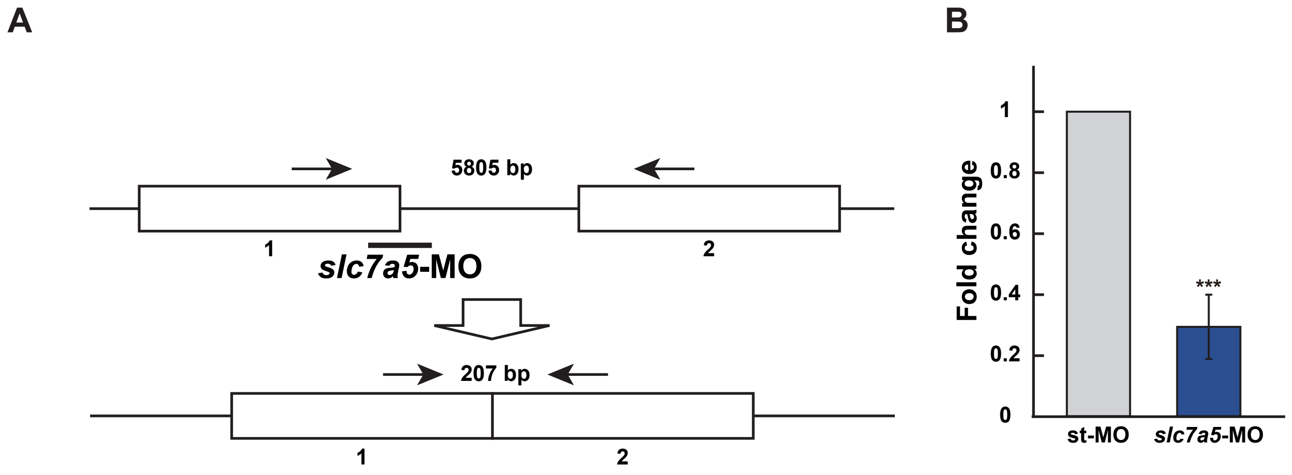
A
5805 bp
1
2
slc7a5-MO
207 bp
1
2
B
Fold change
1
0.8
0.6
0.4
0.2
0
***
st-MO
slc7a5-MO

Figure S6. ***slc7a5*** **pre-mRNA splicing was blocked by vivo-MO.**

(A) Scheme of the *slc7a5* pre-mRNA regions analyzed for splicing (boxed, exons; lines, introns; arrows, primers). (B) Spliced PCR products of *slc7a5*, obtained by qPCR, were significantly reduced in *slc7a5*-MO-injected fins when compared to in st-MO-injected fins by qPCR. ***$p < 0.001$ by Student's *t* test. Error bars represent the standard error.

Figure S7

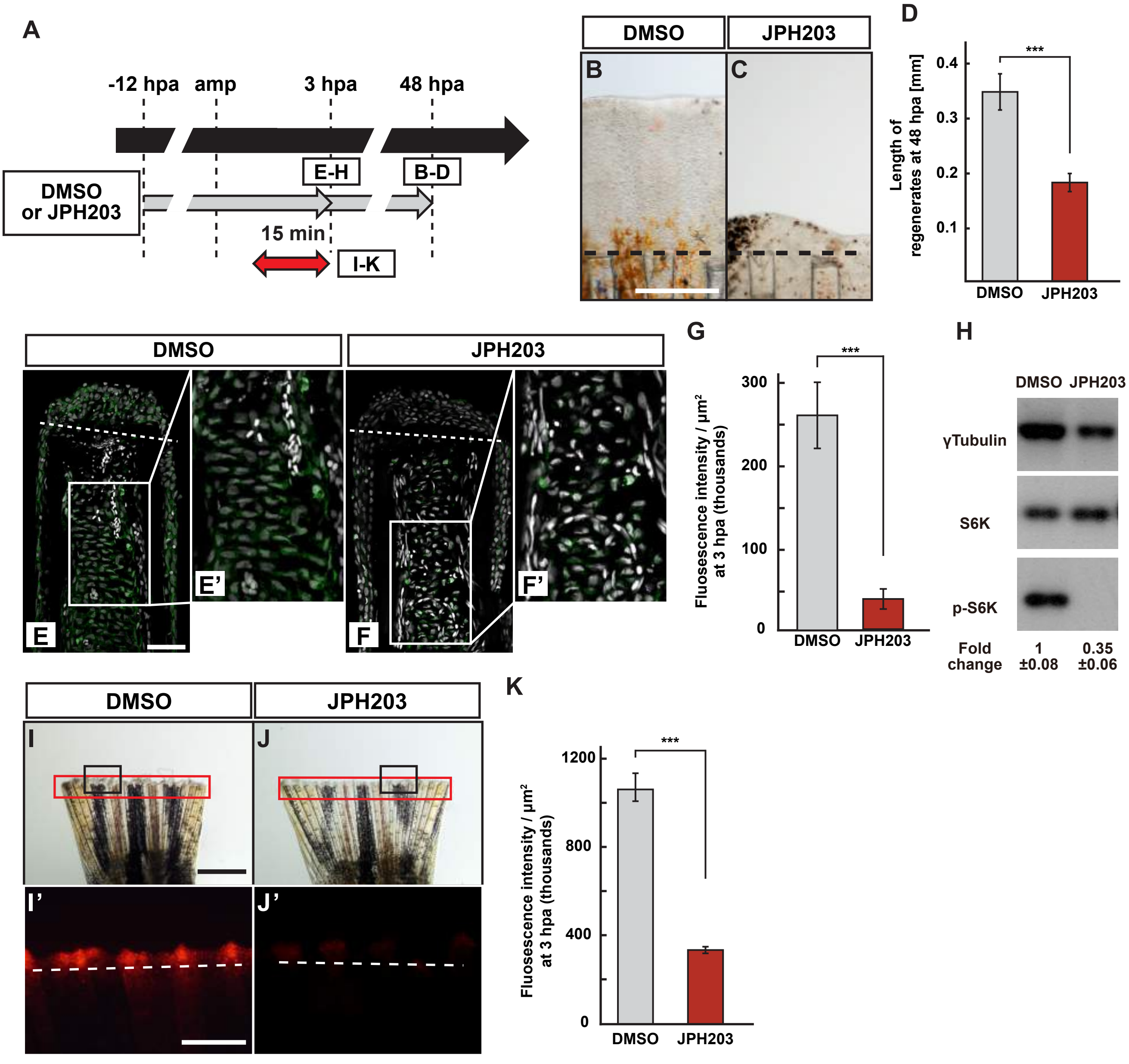
A
-12 hpa
amp
3 hpa
48 hpa
E-H
B-D
DMSO or JPH203
15 min
I-K
DMSO
JPH203
B
C
D
Length of regenerates at 48 hpa [mm]
0.4
0.3
0.2
0.1
***
DMSO
JPH203
E
E'
F
F'
G
Fluosescence intensity / μm² at 3 hpa (thousands)
300
200
100
0
H
DMSO JPH203
γTubulin
S6K
p-S6K
Fold change
1 ±0.08
0.35 ±0.06
I
J
I'
J'
K
1200
800
400

Figure S7. S6K activation and lysosomal acidification are inhibited by JPH203.

(A) Scheme of JPH203 (Slc7a5-specific inhibitor) treatment from -12 to 3 or 48 hpa. A red two-headed-arrow indicates LysoTracker treatment, which was applied 15 min before observation. (B-D) Outgrowth of fin regenerates and quantification of their length in control or JPH203-treated fin regenerates at 48 hpa (n = 5). Representative images (B and C) used for quantification are shown in D. Black dashed lines indicate the amputation planes. Scale bars: 250 μm. ****p* < 0.001 by Student's *t* test. Error bars represent the standard error. (E-G) Longitudinal ray sections and quantification of p-S6K fluorescence intensities per area that consists of the whole regenerates and 500 μm below the amputation plane in DMSO- or JPH203-treated fin regenerates at 3 hpa; p-S6K and nuclei were visualized by immunohistochemical staining and DAPI staining, respectively (n = 5). Representative images (E-F') used for quantification are shown in G. White dashed lines indicate the amputation planes. Scale bars: 50 μm (E and F) and 25 μm (E' and F'). ****p* < 0.001 by Student's *t* test. Error bars represent the standard error. (H) Western blot analysis of γTubulin, S6K, and p-S6K in the DMSO- or JPH203-treated fin stumps (n = 6). γTubulin serves as a loading control. Numbers below each lane show the level of p-S6K in JPH203-treated fin stumps relative to that in DMSO-treated fin stumps at 3 hpa normalized to loading control. (I-K) Images of bright-field and fluorescence microscopy, and quantification of LysoTracker fluorescence intensities in DMSO- or JPH203-treated fins at 3 hpa (n = 8). Black

boxed areas in I and J are enlarged in I' and J', respectively. The LysoTracker fluorescence intensities in red boxed areas were measured (I-J'). Representative images (I'-J') used for quantification are shown in K. White dashed lines indicate the amputation planes. Scale bars: 1 mm (I and J) and 500 μm (I' and J'). ***$p < 0.001$ by Student's *t* test. Error bars represent the standard error.

Figure S8

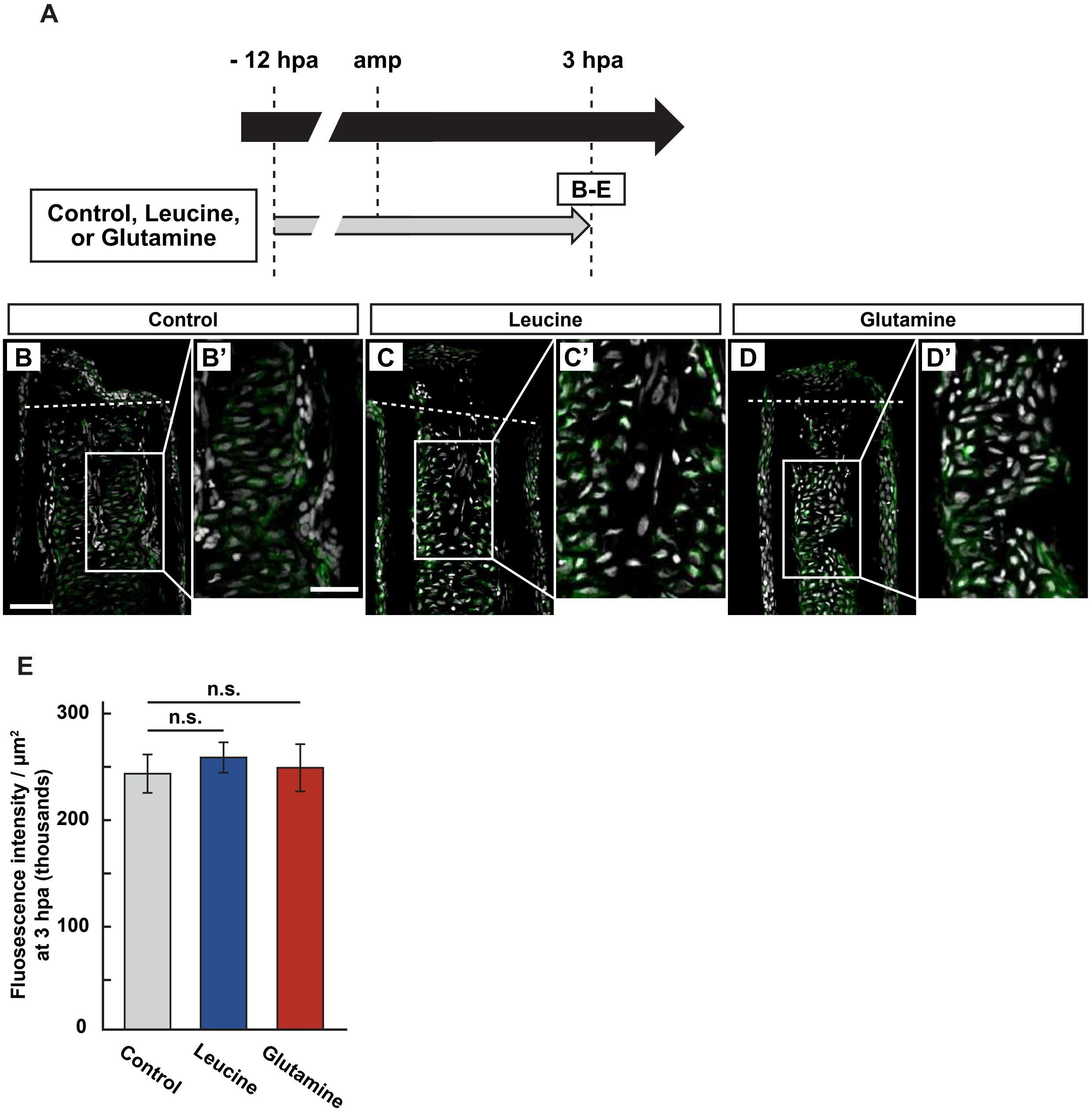
A
- 12 hpa
amp
3 hpa
B-E
Control, Leucine, or Glutamine
Control
Leucine
Glutamine
B
B'
C
C'
D
D'
E
n.s.
n.s.
300
200
100
0
Fluosescence intensity / μm²
at 3 hpa (thousands)
Control
Leucine
Glutamine

Figure S8. Leucine or glutamine treatment has no effect on S6K activation.

(A) Experimental scheme of leucine or glutamine treatment from -12 to 3 hpa. (B-E) Longitudinal ray sections and quantification of p-S6K fluorescence intensities per area that consists of the whole regenerates and 500 μm below the amputation plane in control, leucine-, or glutamine-treated fin regenerates at 3 hpa; p-S6K and nuclei were visualized by immunohistochemical staining and DAPI staining, respectively (n = 5). Representative images (B-D') used for quantification are shown in E. White dashed lines indicate the amputation planes. Scale bars: 50 μm. n.s.: not significant. Error bars represent the standard error.

Figure S9

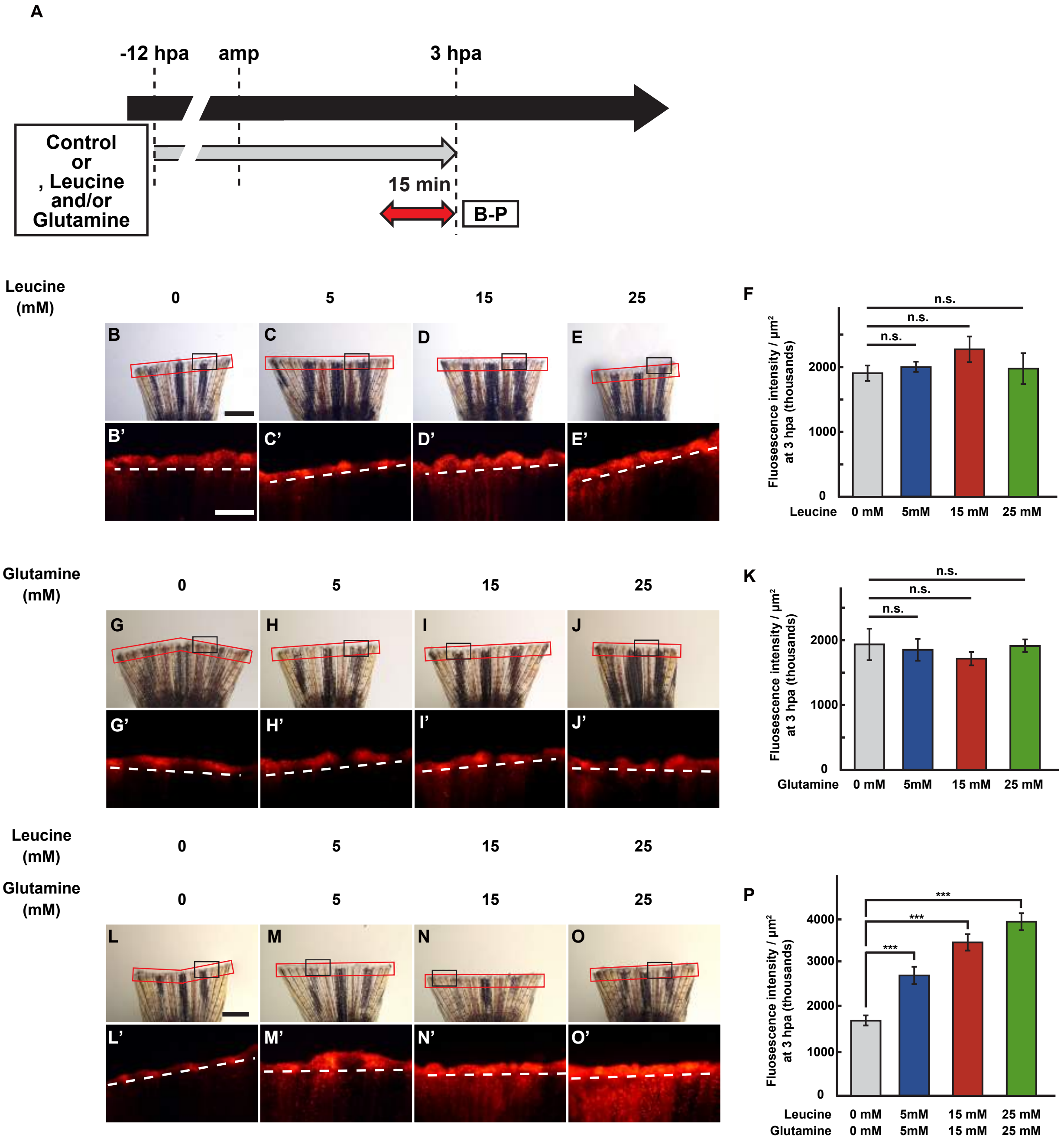
A
-12 hpa
amp
3 hpa
Control
or
, Leucine
and/or
Glutamine
15 min
B-P
Leucine
(mM)
0
5
15
25
B
C
D
E
B'
C'
D'
E'
F
n.s.
n.s.
n.s.
Fluosescence intensity / μm²
at 3 hpa (thousands)
2000
1000
0
Leucine
0 mM
5mM
15 mM
25 mM
Glutamine
(mM)
0
5
15
25
G
H
I
J
G'
H'
I'
J'
K
n.s.
n.s.
n.s.
Fluosescence intensity / μm²
at 3 hpa (thousands)
2000
1000
0
Glutamine
0 mM
5mM
15 mM
25 mM
Leucine
(mM)
0
5
15
25
Glutamine
(mM)
0
5
15
25
L
M
N
O
L'
M'
N'
O'
P
***
***
***
Fluosescence intensity / μm²
at 3 hpa (thousands)
4000
3000
2000
1000
0
Leucine
0 mM
5mM
15 mM
25 mM
Glutamine
0 mM
5mM
15 mM
25 mM

Figure S9. LG treatment up-regulates lysosomal acidification in a concentration-dependent manner.

(A) Experimental scheme of leucine and/or glutamine treatment from -12 to 3 hpa. A red two-headed-arrow indicates LysoTracker treatment, which was applied 15 min before observation. (B-P) Images of bright-field and fluorescence microscopy, and quantification of LysoTracker fluorescence intensities in fin stumps at 3 hpa (n = 6). Black boxed areas in B-E, G-J, and L-O are enlarged in B'-E', G'-J', and L'-O', respectively. The LysoTracker fluorescence intensities in red boxed area were measured (B-E', G-J', and L-O'). Representative images (B-E', G-J', and L-O') used for quantification are shown in F, K, and P. White dashed lines indicate the amputation planes. Scale bars: 1 mm (B-E, G-J, and L-O) and 500 μm (B'-E', G'-J', and L'-O'). ****p* < 0.001 by Student's *t* test. Error bars represent the standard error.

Figure S10

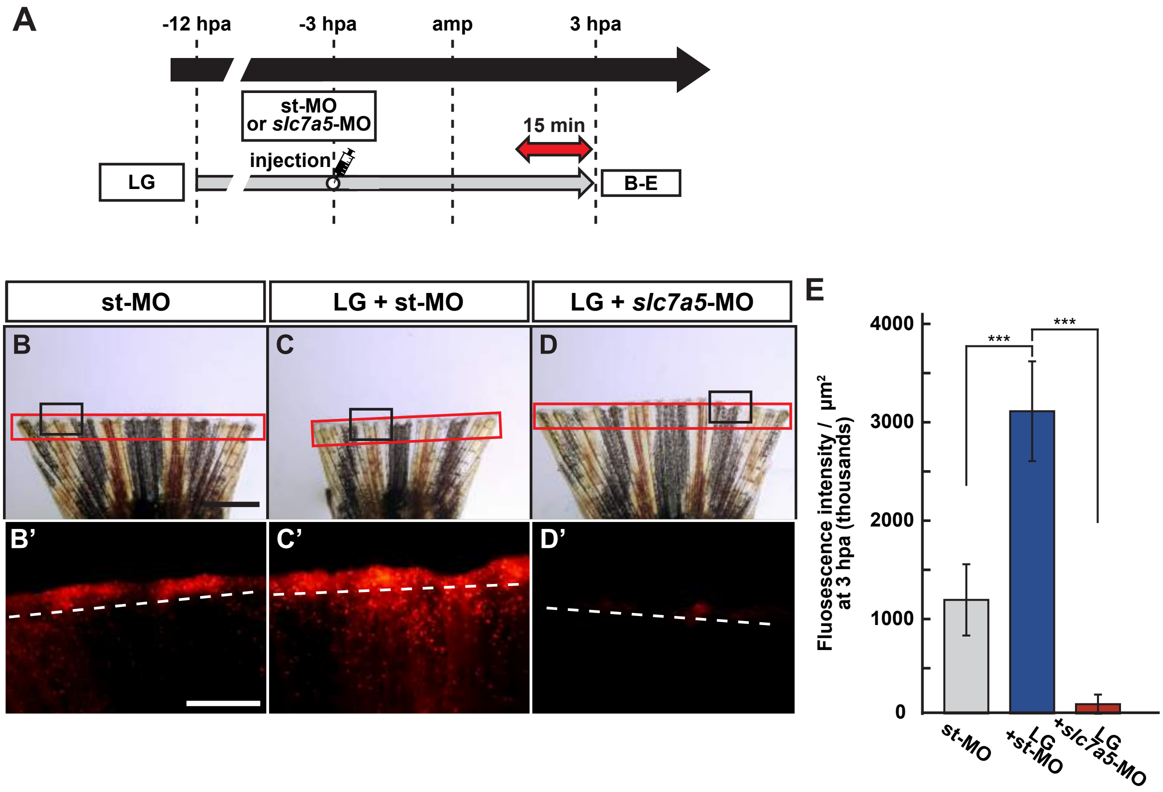
A
-12 hpa
-3 hpa
amp
3 hpa
st-MO
or slc7a5-MO
injection
15 min
LG
B-E
st-MO
LG + st-MO
LG + slc7a5-MO
B
C
D
B'
C'
D'
E
Fluosescence intensity / μm²
at 3 hpa (thousands)
4000
3000
2000
1000
0
***
***
st-MO
LG
+st-MO
LG
+slc7a5-MO

Figure S10. Upregulation of lysosome acidification by LG treatment is blocked by Slc7a5 knockdown.

(A) Experimental scheme of LG treatment from -12 to 3 hpa. A red two-headed-arrow indicates LysoTracker treatment, which was applied 15 min before observation. A syringe indicates vivo-MO injection at -3 hpa. (B-D’) Images of bright-field and fluorescence microscopy, and quantification of LysoTracker fluorescence intensities in st-MO-, LG-treated and st-MO-injected (LG + st-MO), or LG-treated and *slc7a5*-MO-injected (LG + *slc7a5*-MO) fin stumps at 3 hpa (n = 6). Black boxed areas in B-D are enlarged in B’-D’, respectively. The LysoTracker fluorescence intensities in red boxed areas were measured (B-D’). Representative images (B’-D’) used for quantification are shown in E. Scale bars: 1 mm (B-D) and 500 μm (B’-D’). ***$p < 0.001$. Error bars represent the standard error.

Figure S11

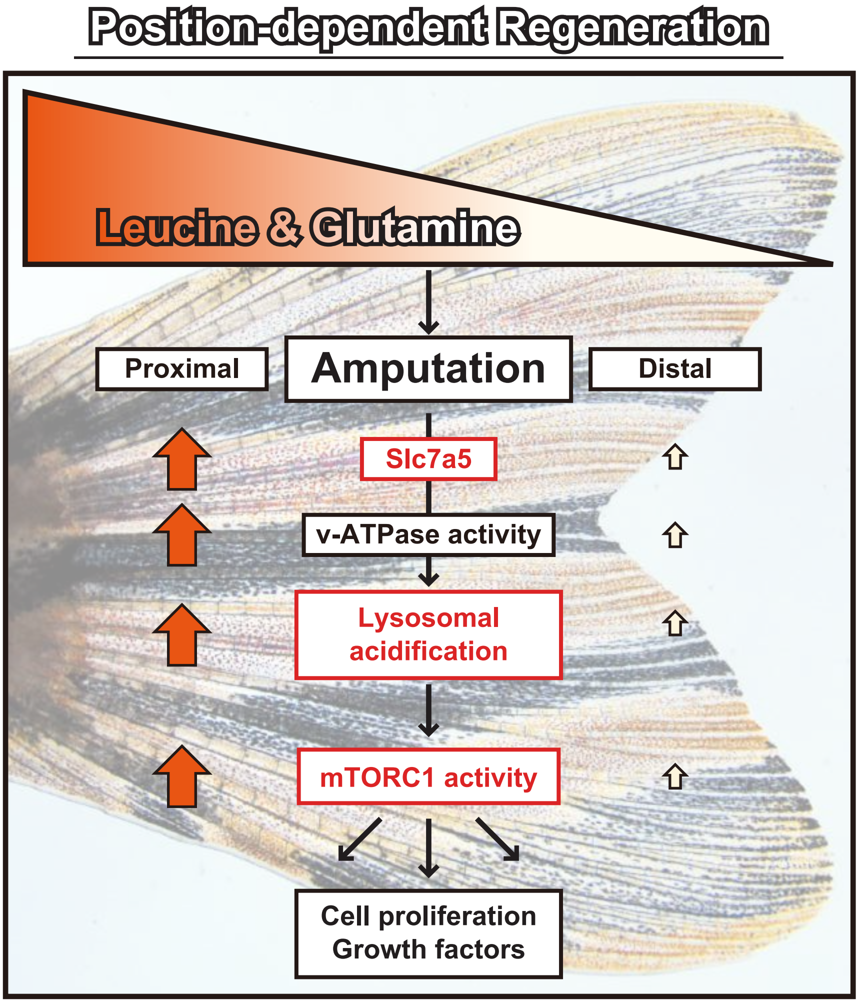
Position-dependent Regeneration
Leucine & Glutamine
Proximal
Amputation
Distal
Slc7a5
v-ATPase activity
Lysosomal acidification
mTORC1 activity
Cell proliferation
Growth factors

Figure S11. A proposed model of position-dependent fin regeneration in zebrafish. For discussion, see text. Leucine and glutamine are proximally enriched in unamputated fins. The position-dependency of *slc7a5* expression, lysosomal acidification, and mTORC1 activity is found in this study (marked by red boxes).

Table S1

	Primer sequences 5' to 3'		
**Gene name**	**Forward primer**	**Reverse primer**	**Ref.**
**qRT-PCR**			
wnt10a	ATTCACTCCAGGATGAGACTTCATA	GTTTCTGTTGTGGGCTTTGATTAG	1
igf2b	GCAGGTCATTCCAGTGATGC	TCTGAGCAGCCTTTCTTTGC	2
aldh1a2	GCTTACCTTGCTACCCTGGAGTC	CAATGGGCTCATGTCTGGTGAG	This study
fgf20a	GGACCACAGCAGATTTGGTATATTGG	CAGCTTTTCAGATCCGTACAGTTCGC	This study
slc7a5	GGAGATCGGCAAAGGTGATAC	GCTCAATCATCTCCTCTGTGAC	This study
slc7a8a	GCAGGGTTTCTGCGATTATGG	CAAGTCAGCAGCAACAAGCAG	This study
slc7a8b	CAGCAAGAAGAGCAGGAATGG	GTTTGGAAGTCTCTCTGTGCC	This study
slc43a1a	CGTAACGTCACGGTTAGCACTT	CTTCACCAGTAGCATTCACTGTC	This study
slc43a1b	GGCAGCATCAGGAGAAGAAGTG	CGAACACAGCAGGTTCTCCAAC	This study
rpl13a	TCTGGAGGACTGTAAGAGGTATGC	AGACGCACAATCTTGAGAGCAG	3
**checking morpholino efficacy**			
slc7a5	TGAAACTGTGGATCGAGCTG	AGGCATCTTGAACCCTTGTG	4

References

1 Bouzaffour, M., Dufourcq, P., Lecaudey, V., Haas, P. & Vriz, S. Fgf and Sdf-1 pathways interact during zebrafish fin regeneration. *PLoS One* **4**, e5824, doi:10.1371/journal.pone.0005824 (2009).

2 Chablais, F. & Jazwinska, A. IGF signaling between blastema and wound epidermis is required for fin regeneration. *Development* **137**, 871-879, doi:10.1242/dev.043885 (2010).

3 Hirose, K., Shimoda, N. & Kikuchi, Y. Transient reduction of 5-methylcytosine and 5-hydroxymethylcytosine is associated with active DNA demethylation during regeneration of zebrafish fin. E*pigenetics* 8**,** 899-906, doi:10.4161/epi.25653 (2013).

4 Chung, J. *et al.* The mTORC1/4E-BP pathway coordinates hemoglobin production with L-leucine availability. S*ci Signal* 8**,** ra34, doi:10.1126/scisignal.aaa5903 (2015).
